# Small Stokes Shift Induced Highly Efficient and Thermally Stable Broadband Near‐Infrared Antimonite Double Perovskite Emitters for Spectroscopy Applications

**DOI:** 10.1002/advs.202509583

**Published:** 2025-07-12

**Authors:** Zhihao Zhou, Hongjun Jiang, Bozhao Yin, Guocheng Ji, Enhai Song, Jianrong Qiu, Zhongmin Yang, Guoping Dong

**Affiliations:** ^1^ State Key Laboratory of Luminescent Materials and Devices Guangdong Provincial Key Laboratory of Fiber Laser Materials and Applied Techniques School of Materials Science and Engineering South China University of Technology Guangzhou 510641 China; ^2^ School of Optoelectronic Engineering Guangdong Polytechnic Normal University Guangzhou 510665 China; ^3^ State Key Laboratory of Modern Optical Instrumentation, College of Optical Science and Engineering Zhejiang University Hangzhou 310027 China

**Keywords:** Cr^3+^ ion, high efficiency, near‐infrared emission, Stokes shift, thermal stability

## Abstract

The exploration of efficient broadband portable near‐infrared (NIR) light sources is crucial for next‐generation NIR spectroscopy‐based technologies. However, developing thermally stable and highly efficient NIR photonic materials exceeding 830 nm is met with limited success. Here, a series of broadband NIR phosphors with long‐wavelength emission (λ_em_ > 830 nm) is designed by incorporating activator Cr^3+^ ions into ALaMgSbO_6_ (A = Ca, Sr) double perovskite matrices. Specifically, a cation site substitution strategy is proposed to reduce the Stokes shift of ALaMgSbO_6_:Cr^3+^ (A = Ca, Sr), rendering these as‐prepared NIR phosphors possess excellent thermal resistance performance (89.80%@423 K) and high quantum efficiency (82.5%) simultaneously. Structural analyses, DFT calculations, and spectroscopy measurements revealed that Cr^3+^ ions can occupy both [SbO_6_] and [MgO_6_] polyhedral sites but prefer to replace Sb^5+^ ions in ALaMgSbO_6_ (A = Ca, Sr). The luminescence efficiency and thermal stability of the samples are further improved through a flux strategy, and the emission spectra are effectively broadened by the introduction of Yb^3+^ as an extra NIR emitter. Furthermore, the designed phosphors exhibit a full visible‐spectrum conversion ability from 400 to 800 nm, showing great promise for versatile NIR spectroscopy applications in solar energy harvesting, night vision, non‐destructive visualization, and dental analysis.

## Graphical abstract

Highly efficient and thermally stable NIR photonic materials with emission over 830 nm are designed. A cation site substitution strategy is proposed to regulate the Stokes shift of the NIR emitters. The phosphors exhibit a full visible‐spectrum conversion ability from 400 to 800 nm, showing great promise for NIR spectroscopy applications in solar energy harvesting, night vision, and non‐destructive visualization.

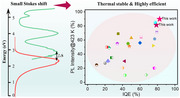

## 1 Introduction

Due to the advantages of non‐destructive and instant recognition with high sensitivity and spatiotemporal resolution, near‐infrared (NIR) light sources have extensive applications in fields of night vision, plant growth, NIR spectral analysis, medical imaging and diagnostics, optical communication, etc.^[^ ^1^, ^2^, ^3^, ^4^, ^5^, ^6^ ^]^ With the emerging popularity of intelligent facilities, substantial efforts have been focused in the past ten years to explore portable and miniaturization NIR light sources that are capable of integrating into smartphones for multifunctional applications.^[^ ^7^, ^8^ ^]^ Consequently, it is imperative to develop photonic materials that can effectively transform full visible light into broadband NIR emission for constructing smart optical devices. The star dopant, transition metal Cr^3+^ ion, has aroused considerable attention owing to its abundance of energy levels, large absorption cross‐section, high luminescence efficiency, and tunable emission wavelength.^[^ ^9^, ^10^, ^11^ ^]^ A large number of broadband NIR luminescent materials have been designed by doping Cr^3+^ into suitable matrices, including garnet, spinel, pyroxene, phosphate, borate, fluoride, and so on.^[^ ^12^, ^13^, ^14^, ^15^, ^16^, ^17^, ^18^, ^19^, ^20^, ^21^ ^]^ Among them, the Cr^3+^‐activated garnet phosphors were most widely investigated, achieving high efficiency and thermal quenching resistance of NIR emissions.^[^ ^22^, ^23^, ^24^, ^25^, ^26^ ^]^ Nevertheless, the emission peaks of these relevant garnet‐based NIR phosphors are severely constrained within 830 nm because of the compacted atomic arrangement, which will greatly hinder the practical spectroscopy applications. Hence, the exploration of suitable host material for activating Cr^3+^ emission is crucial to effectively customize a new kind of efficient long‐wavelength emission NIR materials.

Double perovskite oxides (AA′BB′O_6_) have recently attracted increasing interest owing to their excellent composition flexibility and stability of chemical and physical properties. In this crystal structure, the B/B′ sites coordinate with six oxygen forming abundant [BO_6_]/[B′O_6_] octahedra, which can provide appropriate occupation sites for Cr^3+^ ions to realize NIR spectral tuning.^[^ ^27^ ^]^ At present, a series of Cr^3+^‐doped double perovskite photonic materials have been reported, which can easily achieve NIR emission over 830 nm. Despite considerable efforts, progress in the search for long‐wavelength NIR luminescent materials with both high luminescence efficiency and thermal stability is still limited (Figure S1, Supporting Information).^[^ ^28^, ^29^, ^30^, ^31^ ^]^ The Stokes shift theory proposed by G. Blasse can predict the luminescence efficiency and thermal stability of phosphors, although the influence of the Stokes shift has been underestimated in the past few decades.^[^ ^32^ ^]^ Wen et al. also revealed that a small Stokes shift is usually accompanied by high luminescence efficiency and thermal stability through the DFT calculation method.^[^ ^33^ ^]^ Zhang et al. reduced the Stokes shift from 2990 to 2854 cm^−1^ by chemical unit con‐substitution of Lu^3+^‐Al^3+^ for Ca^2+^‐Zr^4+^ in Ca_2_LuZr_2_Al_3_O_12_:Cr^3+^ phosphor, resulting in a significant improvement in quantum efficiency and thermal anti‐quenching property.^[^ ^34^ ^]^ Therefore, reducing the Stokes shift to suppress the non‐radiative transition can be an effective strategy for constructing long‐wavelength, highly efficient NIR emission phosphors.

Herein, Ca/SrLaMgSbO_6_ (abbreviated as CLMSO and SLMSO) double perovskite oxides were selected to accommodate Cr^3+^ ions for acquiring efficient broadband NIR phosphors with long‐wavelength emission (λ_em_ > 830 nm). A structural modulation strategy is proposed through the cation substitution of A‐B sites to reduce the Stokes shift of ALMSO:Cr^3+^ (A = Ca, Sr), making these designed NIR phosphors possess high quantum efficiency (82.5%) and excellent thermal stability (89.80%@423 K) simultaneously. The detailed site occupation of Cr^3+^ in ALMSO (A = Ca, Sr) is unravelled by structural analyses, DFT calculations, and spectroscopy measurements, which confirms that Cr^3+^ ions occupy both [SbO_6_] and [MgO_6_] polyhedral sites but prefer to enter the Sb^5+^ site. The emission intensity and thermal quenching resistance of the phosphors are further enhanced through a flux strategy, while the emission spectra are effectively broadened by constructing Cr^3+^ → Yb^3+^ energy transfer channel. Moreover, the as‐prepared samples demonstrated a full visible‐spectrum conversion ability from 400 to 800 nm, rendering ALMSO:Cr^3+^, Yb^3+^ (A = Ca, Sr) phosphors highly promising as light conversion materials for solar energy harvesting. The constructed prototype NIR phosphor‐converted light emitting diodes (pc‐LED) devices have a substantial NIR output power of 106.3 mW@400 mA and photoelectric conversion efficiency of 16.08%@20 mA, enabling applied to versatile NIR spectroscopy applications, including night vision, non‐destructive visualization, and dental analysis.

## 2 Results and Discussion

### 2.1 Screening of Hosts, Crystal Structure, and Microstructure Analysis

**Figure 1:**
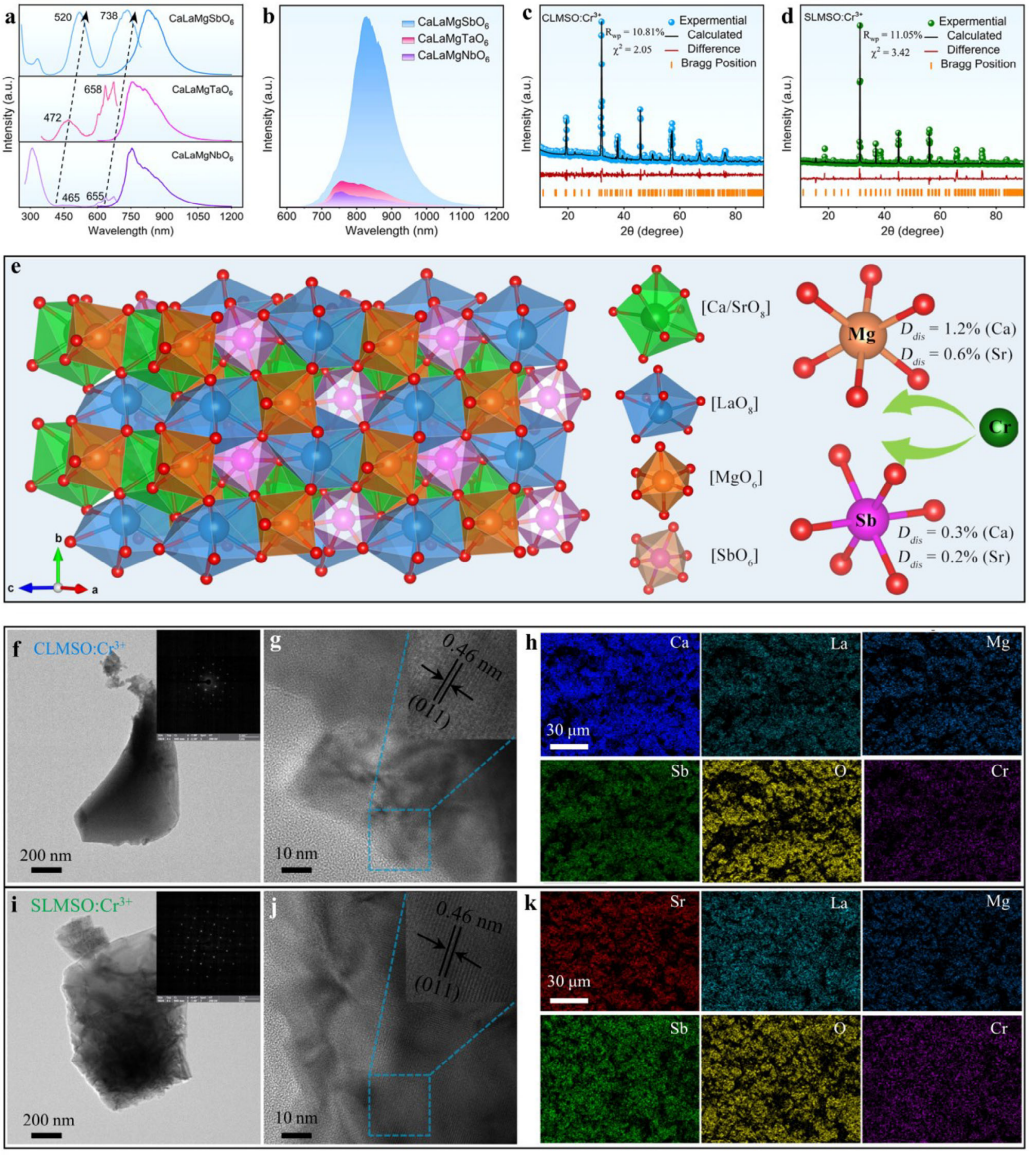
a) Normalized PLE and PL spectra of CaLaMgXO_6_:Cr^3+^ (X = Nb, Ta, Sb). b) Variation trend of PL spectra in CaLaMgXO_6_:Cr^3+^ (X = Nb, Ta, Sb) phosphors. c,d) XRD Rietveld refinements of ALaMgSbO_6_:Cr^3+^ (A = Ca, Sr) samples. e) Crystal structure of ALaMgSbO_6_ (A = Ca, Sr) compounds. f,i) TEM images, g,j) HRTEM images, and h,k) elemental mapping images of ALaMgSbO_6_:Cr^3+^ (A = Ca, Sr) samples.

Choosing the best combination of elements from ALaMgXO_6_ (A = Ca, Sr; X = Nb, Ta, Sb) double perovskite family to obtain efficient Cr^3+^‐activated NIR phosphors was first investigated through the structural modulation of A and X sites. The normalized photoluminescence excitation (PLE) and emission (PL) spectra of CaLaMgXO_6_:Cr^3+^ (X = Nb, Ta, Sb) phosphors are given in **Figure** **1**a. A tendency of gradual red shift can be observed from the PLE spectra when the X‐site ion varies from Nb^5+^ or Ta^5+^ to Sb^5+^, where the excitation wavelength of these Cr^3+^‐activated phosphors can be effectively tuned from 465 to 520 nm (take the blue‐green visible region as an example). The gradual red shift of the primary excitation band from the blue region to the green region is beneficial in reducing blue light hazards. Moreover, the PL intensity progressively enhances as the X‐site ion alters from Nb^5+^ and Ta^5+^ to Sb^5+^, which may be ascribed to the reduced probability of non‐radiative transition owing to the Stokes shift gradually decreasing (Figure 1b). To further optimize the optical properties, the A‐A′ sites were replaced with alkaline earth and rare earth ions via equivalent cationic substitution. Introducing cations with Ca‐La, Ca‐Gd, and Sr‐La at the A‐A′ sites in AA′MgSbO_6_ compounds can gain the optimal NIR emission performance (Figure S1, Supporting Information). In consideration of the Stokes shift along with the PL intensity of ALaMgXO_6_:Cr^3+^ (A = Ca, Sr; X = Nb, Ta, Sb) samples, the ALaMgSbO_6_:Cr^3+^ (A = Ca, Sr) phosphors were selected for further study.

X‐ray diffraction (XRD) patterns of Cr^3+^‐doped ALaMgSbO_6_ (A = Ca, Sr) samples (abbreviated as CLMSO:Cr^3+^ and SLMSO:Cr^3+^) with different doping concentrations are well consistent with the standard card, confirming that pure phases were formed and Cr^3+^ ions have successfully incorporated into CLMSO and SLMSO matrixes (Figure S2, Supporting Information). To verify the actual crystallographic characteristics of the obtained phosphors, Rietveld structural refinement was conducted on ALMSO (A = Ca, Sr) matrices (Figure S3, Supporting Information) and the representative Cr^3+^‐doped samples (Figure 1c,d). The detailed refinement parameters and crystal structure information are presented in Tables S2 and S3, Supporting Information. The low convergence factor values of *R_wp_* = 10.81%, χ^2^ = 2.05 for CLMSO:Cr^3+^ and *R_wp_* = 11.05%, χ^2^ = 3.42 for SLMSO:Cr^3+^ demonstrated the reliability of the refined results. According to the refined results, all the observed XRD peaks match well with the simulated XRD peaks, further proving the formation of pure phases. The exact crystal structure of ALMSO (A = Ca, Sr) compounds, as depicted in Figure 1e, belongs to the monoclinic crystal system *P2_1_/n* space group. The Mg^2+^ and Sb^5+^ ions occupy the B‐B′ sites, forming alternate and vertex‐shared [MgO_6_] and [SbO_6_] octahedrons arranged to compose the crystal network structure, while the A‐A′ sites Ca^2+^ and La^3+^ ions are coordinated with eight oxygen atoms forming [CaO_8_]/[LaO_8_] dodecahedron to maintain structural stability. According to the Hume‐Rother rule, it is supposed that Cr^3+^ ions (r_CN‐6_ = 0.615 Å) are likely to replace Sb^5+^ (r_CN‐6_ = 0.6 Å) or Mg^2+^ (r_CN‐6_ = 0.72 Å), or occupy both Sb^5+^ and Mg^2+^ lattice sites in theory (Equation S1, Supporting Information).^[^ ^35^ ^]^ The cell volumes of Cr^3+^‐doped samples are slightly larger than those of the ALMSO (A = Ca, Sr) hosts, which suggests that Cr^3+^ ions may be more inclined to enter the Sb^5+^ site with a smaller ionic radius. But the specific site occupation needs to be further discussed in detail in the subsequent analysis.

Scanning electron microscopy (SEM) images show that the ALMSO:Cr^3+^ (A = Ca, Sr) phosphors consist of irregular polygonal particles with sizes of several microns (Figure S4, Supporting Information). In addition, high‐resolution transmission electron microscopy (HRTEM) and selected area electron diffraction (SAED) images of single particles were also performed. As shown, regular diffraction spots and smooth particle surfaces can be detected, essentially manifesting the good crystallinity characteristic of ALMSO:Cr^3+^ (A = Ca, Sr) samples (Figures 1f,i). The lattice spacing measured from HRTEM images is 0.46 nm, which can be assigned to the (011) plane of ALMSO (A = Ca, Sr) double perovskite oxides (Figure 1g,j). Elemental mapping images, as presented in Figure 1h,k, reveal that all the elements Ca/Sr, La, Mg, Sb, O, and Cr are uniformly distributed in the selected area. Additionally, EDS results demonstrate that the atomic ratio of the elements in ALMSO:Cr^3+^ (A = Ca, Sr) is close to the nominal stoichiometric ratio (Figure S5, Supporting Information).

### 2.2 Photoluminescence Properties and Site Occupancy Analysis

Two characteristic peaks at ≈573.8 and 584.1 eV associated with the spin‐orbit splitting of Cr^3+^ can be detected in high‐resolution X‐ray photoelectron spectroscopy (XPS) spectra, implying that the actual valence state of chromium ion is +3 in ALMSO:Cr^3+^ (A = Ca, Sr) (Figure S6, Supporting Information).^[^ ^36^ ^]^ The Cr^3+^‐doped samples exhibit obvious absorption in ultraviolet, green, and red regions compared to the ALMSO (A = Ca, Sr) hosts, and thus, the body color of the samples varies from white to pink after Cr^3+^ ion doping. Besides, the diffuse reflection (DR) spectra have no absorption in the region of 800–1400 nm, further excluding the existence of Cr^4+^ in the as‐prepared samples. The band gap of ALMSO (A = Ca, Sr) is calculated to be 4.85 and 4.78 eV, respectively, which can provide sufficient energy space to accommodate the dopants (Figure S7, Supporting Information). The larger band gap manifests that Cr^3+^‐doped ALMSO (A = Ca, Sr) phosphors will not only have favorable conditions to achieve excellent NIR emission properties but also remarkable resistance to the thermal ionization process.^[^ ^37^ ^]^

**Figure 2:**
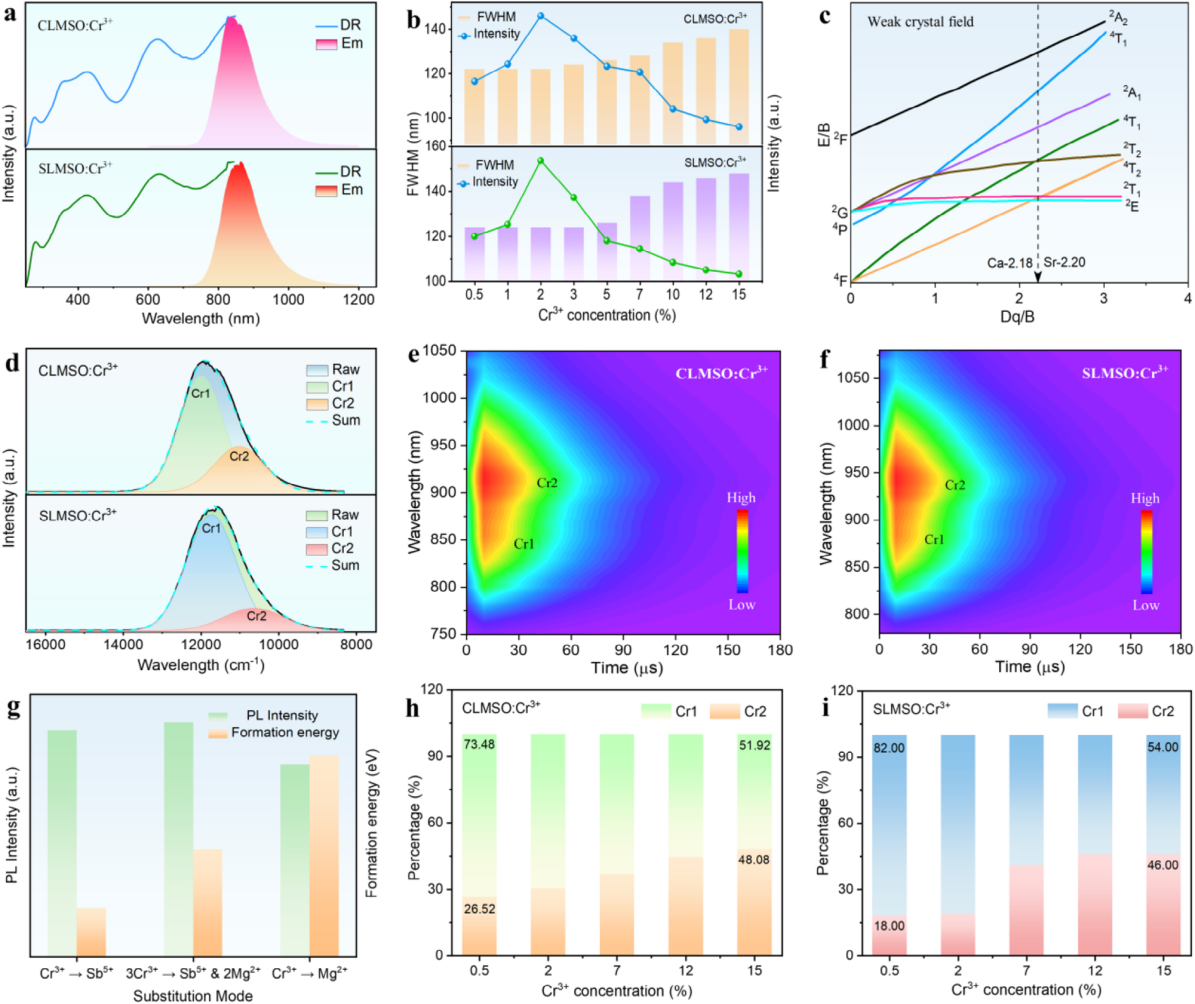
a) Normalized DR and PL spectra of ALMSO:Cr^3+^ (A = Ca, Sr) phosphors. b) Variation of PL intensity and FWHM in ALMSO:Cr^3+^ (A = Ca, Sr) as a function of Cr^3+^ contents. c) Tanable‐Sugnao diagram for Cr^3+^ in octahedral coordination. d) Gaussian Fitting of PL spectra, and e,f) TRPL mapping of ALMSO:Cr^3+^ (A = Ca, Sr). g) Formation energy analysis and corresponding PL intensity of Cr^3+^ doped at different substitution modes. h‐i) The Cr1 and Cr2 ratio changes with Cr^3+^ concentrations in ALMSO:Cr^3+^ (A = Ca, Sr) samples.

ALMSO:Cr^3+^ (A = Ca, Sr) possesses three characteristic excitation bands covering from the UV to the NIR region. The ultraviolet excitation at 330 nm should be assigned to the overlap of host absorption and ^4^A_2_ → ^4^T_1_ (^4^P) transition of Cr^3+^,^[^ ^38^, ^39^ ^]^ while the other dominant absorption bands located at 520 and 740 nm are corresponding to the *d–d* transitions of Cr^3+^ from ^4^A_2_ → ^4^T_1_ (^4^F) and ^4^A_2_ → ^4^T_2_ (^4^F), respectively (**Figure** **2**a). Upon light excitation at 520 nm, ALMSO:Cr^3+^ (A = Ca, Sr) exhibits a broadband NIR emission peaking at 836 (CLMSO:Cr^3+^) and 866 nm (SLMSO:Cr^3+^), which stem from the ^4^T_2_ (^4^F) → ^4^A_2_ transition of Cr^3+^ in weak crystal field.^[^ ^40^ ^]^ The redshift of excitation peak of ALMSO:Cr^3+^ (A = Ca, Sr) can be clearly observed when compared with other reported Cr^3+^‐activated NIR phosphors, suggesting that the developed phosphors have a small Stokes shift. The Stokes shift (ΔS) of ALMSO:Cr^3+^ (A = Ca, Sr) samples is calculated to be 1625.24 (CLMSO:Cr^3+^) and 1966.17 cm^−1^ (SLMSO:Cr^3+^), respectively, which is much smaller than other NIR phosphors such as ZnTa_2_O_6_:Cr^3+^ (4276 cm^−1^), LiIn_2_SbO_6_:Cr^3+^ (3977 cm^−1^), LiGaP_2_O_7_:Cr^3+^ (3500 cm^−1^), etc.^[^ ^37^, ^41^, ^42^, ^43^ ^]^ Therefore, the developed phosphors are capable of effectively restraining the non‐radiative transition and allowing most of the excited photons to return to the ground state, thus a higher luminescence efficiency than other NIR phosphors with long emission wavelength (λ_em_ > 800 nm) can be expected. The PL intensity of ALMSO:Cr^3+^ (A = Ca, Sr) reaches the maximum value when the doping concentration of Cr^3+^ is 2%, and then gradually decreases with higher Cr^3+^ concentration due to the concentration quenching effect (Figure 2b; Figure S8, Supporting Information). The interaction coefficient θ is evaluated to be closer to 3, meaning that the concentration quenching mechanism of ALMSO:Cr^3+^ (A = Ca, Sr) is caused by the energy transfer between the nearest Cr^3+^ ions (Figure S9, Supporting Information).^[^ ^44^ ^]^ The emission spectra exhibit a slight red shift and the full‐width at half maximum (FWHM) value increase gradually with the doping concentration increase while the excitation spectra are almost unchanged, which may be related to the occupation tendency of Cr^3+^ ions in different sites of the crystal structure that will be discussed later (Figure 2b; Figure S10, Supporting Information).

Generally, the optical properties of Cr^3+^ are highly influenced by the crystal field intensity and can be described by the Tanabe‐Sugano diagram (Figure 2c). To reveal the crystal field strength of Cr^3+^ in ALMSO (A = Ca, Sr) matrices, the crystal field strength parameter *Dq* and Racah parameter *B* were calculated by the following equations^[^ ^45^ ^]^:

(1)

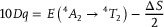

(2) $$\frac{Dq}{B}=\frac{15(x-8)}{x^{2}-10x}$$

(3)

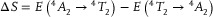



(4)

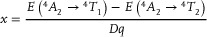




As shown by the parameters provided in Table S4, the crystal field strength *Dq*/*B* of ALMSO:Cr^3+^ (A = Ca, Sr) phosphors was calculated to be 2.18 and 2.20, respectively, implying that the Cr^3+^ ions are located in a weak octahedral coordination environment. Besides, the crystal field strength of the samples only presents a minor change with the increase of doping concentration, which indicates that the local crystal field environment is not significantly affected by the Cr^3+^ contents (Table S5, Supporting Information).

The emission spectra of ALMSO:Cr^3+^ (A = Ca, Sr) exhibit inhomogeneous peak broadening, which can be well‐fitted by Gaussian function (Figure 2d). This implies that Cr^3+^ ions occupy two different lattice sites in the phosphors and form two luminescent centers. The site occupation of Cr^3+^ is also analyzed on the basis of luminescence decay, as performed in the time‐resolved photoluminescence (TRPL) spectra (Figure 2e,f). Two distinct luminescence decay centers can be detected, indicating that they originate from two significantly different luminescence sites (Cr1 and Cr2) in ALMSO:Cr^3+^ (A = Ca, Sr). Based on the aforementioned structural analysis, it is reasonably inferred that Cr^3+^ occupies both the [SbO_6_] and [MgO_6_] octahedral sites to exhibit two different luminescence centers. To assign the exact luminescence centers of ALMSO:Cr^3+^ (A = Ca, Sr) phosphors, the crystal field splitting energy of Cr^3+^ in SbO_6_ and MgO_6_ sites was evaluated by the following formula:^[^ ^46^ ^]^

(5) $$D_{q}=\frac{ze^{2}r^{4}}{6R^{5}}$$

where z is the valence of the anion, *e* represents the charge of the electron, *r* equals the radius of the doping ions, and *R* denotes the distance between the central ion and the associated ligands. The *Dq* value is inversely proportional to *R* ^5^. Considering the radius relationship between Sb^5+^ (r_CN‐6_ = 0.6 Å) and Mg^2+^ (r_CN‐6_ = 0.72 Å), the high‐energy (Cr1) and low‐energy emission peaks (Cr2) can be ascribed to the occupation of Cr^3+^ in [SbO_6_] and [MgO_6_] sites, respectively.

To confirm the substitution tendency of Cr^3+^ in [SbO_6_] and [MgO_6_] sites of the samples, the formation energy (E_form_) of Cr^3+^ in different substitution modes was calculated (Equation S2, Supporting Information). The formation energy of one Cr atom at Sb^5+^ octahedral site was determined to be ‐11.75 eV, which is much lower than the other two substitution modes. In addition, we also experimentally explored the above three substitution modes of CLMSO:Cr^3+^ by controlling the original stoichiometric ratio of starting materials. The result demonstrates that the strategy of Cr^3+^ → Sb^5+^ and 3Cr^3+^ → Sb^5+^ + 2Mg^2+^ substitutions has the highest PL intensity (Figure 2g; Figure S11, Supporting Information). Therefore, the Cr^3+^ dopants have the probability and tendency to occupy both the Sb^5+^ and Mg^2+^ sites in ALMSO (A = Ca, Sr), but Cr^3+^ ions prefer to mainly substitute the Sb^5+^ octahedron site. Multiple emitting centers of Cr^3+^ and their interaction were further verified by Gaussian fitting of the PL spectra with variation of doping concentrations.^[^ ^47^ ^]^ Figures S12, S13 (Supporting Information) give the Cr^3+^ concentration‐dependent Gaussian fitting of the PL spectra in ALMSO:Cr^3+^ (A = Ca, Sr) samples. Obviously, the ratios of Cr1 and Cr2 are changed with Cr^3+^ doping concentrations. According to the fitting results, the luminescent contribution from the Cr2 emitting center increases gradually with higher Cr^3+^ content, further confirming the occurrence of energy transfer of Cr1 to the Cr2 center upon doping more Cr^3+^ ions (Figure 2h,i; Table S6, Supporting Information).^[^ ^48^, ^49^ ^]^ In summary, the above results demonstrate that Cr^3+^ ions favor the substitution of both [SbO_6_] and [MgO_6_] polyhedral sites but are more inclined to occupy the [SbO_6_] site in ALMSO (A = Ca, Sr). When the doping concentration is low, the Cr^3+^ ion will first choose to enter the Sb^5+^ site, and thus the Cr1 center contributes more to PL than the Cr2 center. As the content of Cr^3+^ increases, the interaction between the Cr^3+^‐Cr^3+^ pairs will be strengthened, and thus Cr2 center contributes more to PL at higher doping concentrations.

### 2.3 Thermal Stability and Quantum Efficiency

**Figure 3:**
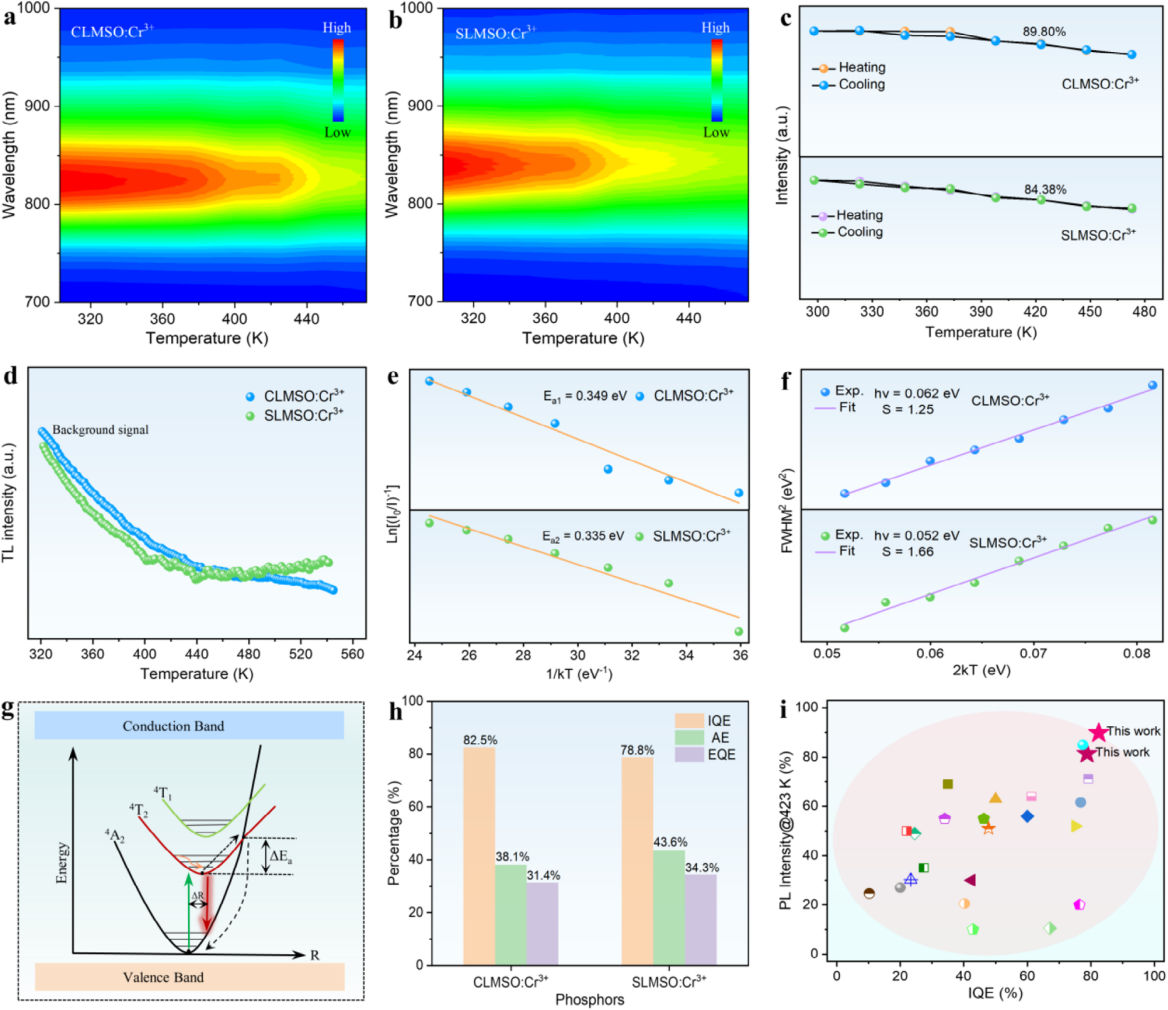
a,b) Temperature‐dependent PL mapping, and c) Integral PL intensity of ALMSO:Cr^3+^ (A = Ca, Sr) phosphors. d) TL curves, and e) Activation energy analysis of ALMSO:Cr^3+^ (A = Ca, Sr). f) Fitting curves of FWHM^2^ versus 2*k*T in ALMSO:Cr^3+^ (A = Ca, Sr). g) Configurational coordinate diagram of the thermal quenching mechanism of ALMSO:Cr^3+^ (A = Ca, Sr). h) Quantum efficiency values of ALMSO:Cr^3+^ (A = Ca, Sr) phosphors. i) Comparison of IQE and thermal stability of ALMSO:Cr^3+^ (A = Ca, Sr) with some other reported NIR phosphors.

To deeply investigate the optical properties of the developed NIR phosphors, the temperature‐dependent PL spectra and mapping plot temperature‐dependent PL spectra are provided to assess the luminescence thermal stability of ALMSO:Cr^3+^ (A = Ca, Sr) samples (**Figure** **3**a,b; Figure S14, Supporting Information). With the temperature, the PL intensity gradually decreases owing to the enhancement of the non‐radiative relaxation process caused by lattice vibration thermal quenching. The corresponding integrated emission intensity acquired from the PL spectra with different temperatures is given in Figure 3c. As shown, the emission intensity at 423 K still can maintain 89.80% (CLMSO:Cr^3+^) and 84.38% (SLMSO:Cr^3+^) of the initial intensity at room temperature, revealing the robust thermal resistance properties of ALMSO:Cr^3+^ (A = Ca, Sr). Also, the emission intensity of the samples remained nearly unchanged after six heating and cooling cycles, exhibiting excellent luminescence reversibility with temperature (Figure S15, Supporting Information).

To explore the influence of defects on the luminescence thermal behavior, thermoluminescence (TL) curves of ALMSO:Cr^3+^ (A = Ca, Sr) were performed. No distinct signals can be detected in the TL curves, which means that there are few trap energy levels in the lattice, and thus the contribution of trap‐assisted emission compensation can be ignored (Figure 3d).^[^ ^50^ ^]^ The activation energy (*ΔE_a_*), which can be an index for analyzing the thermal quenching process, was estimated based on the Arrhenius equation^[^ ^51^ ^]^:

(6) $$I(T)=\frac{I_{0}}{1+A\cdot \exp(-\frac{\Delta E_{a}}{kT})}$$

where *I(T)* and *I_0_* stand for the PL intensity at the given temperature and room temperature, *A* refers to a constant associated with the matrix, and *k* denotes the Boltzmann constant. As depicted in Figure 3e, the activation energy of CLMSO:Cr^3+^ and SLMSO:Cr^3+^ is calculated to be 0.349 and 0.335 eV, respectively. Such a relatively high energy barrier is conducive to retard thermally assisted non‐radiative transition process of this type of Cr^3+^‐activated NIR phosphors with long emission wavelength. To further clarify the origin of the highly thermal stability of the NIR emission in ALMSO:Cr^3+^ (A = Ca, Sr), the strength of the electron–phonon coupling (EPC) effect that can be reflected by the Huang–Rhys factor (*S*) was evaluated:^[^ ^52^ ^]^

(7) $$FWHM=2.36\sqrt{S}\hbar \omega \sqrt{\coth(\frac{\hbar \omega }{2kT})}$$

where *ħω* and *k* are the phonon energy and Boltzmann constant, respectively. According to the fitting results of FWHM^2^ as a function of 2*kT*, the *S* value was determined to be 1.25 (CLMSO:Cr^3+^) and 1.66 (SLMSO:Cr^3+^), as plotted in Figure 3f. Previous studies have shown that a weak EPC effect is in favor of restraining the thermal quenching of the phosphor.^[^ ^53^ ^]^ Therefore, we suppose that the weak EPC effect plays a critical role in the thermal stability of ALMSO:Cr^3+^ (A = Ca, Sr) samples. It is known that the crystal lattices with high Debye temperature (*Θ_D_*) have robust structural rigidity, which can ensure excellent thermal stability, small Stokes shift, and high quantum efficiency of the phosphors.^[^ ^54^ ^]^ The Debye temperature (*Θ_D_*) of ALMSO:Cr^3+^ (A = Ca, Sr) phosphors was calculated to be 475.46 and 465.84 K (Equations S3, S4 and Table S7, Supporting Information), which is higher in comparison to other reported oxide matrixes such as MgAl_2_O_4_ (436 K), Sr_3_NbO_5.5_ (416 K), and YGa_3_(BO_3_)_4_ (400 K), etc.^[^ ^55^, ^56^, ^57^ ^]^ The large *Θ_D_* value corresponds to the weak lattice vibration frequency, which can inhibit the non‐radiative transition, resulting in the remarkable thermal resistance properties of the as‐prepared Cr^3+^‐activated ALMSO (A = Ca, Sr) NIR phosphors.

Furthermore, temperature‐dependent in situ XRD patterns demonstrate no occurrence of impure phase or phase transition even at 823 K, inferring the high structural rigidity of ALMSO (A = Ca, Sr) (Figure S16, Supporting Information). Also, thermogravimetric (TG) analysis reveals that there was no significant weight loss below 873 K of the ALMSO:Cr^3+^ (A = Ca, Sr) phosphors, further confirming the good heat resistance of ALMSO (A = Ca, Sr) compounds (Figure S17, Supporting Information). Besides, the ALMSO (A = Ca, Sr) matrices possess a relatively large bandgap, which is beneficial to suppress the generation of thermal ionization process according to Dorenbos' thermal ionization model theory.^[^ ^58^, ^59^ ^]^ Hence, the thermal cross‐relaxation mechanism is more likely responsible for the luminescence thermal quenching of ALMSO:Cr^3+^ (A = Ca, Sr) phosphors, as demonstrated by the configurational coordinate diagram in Figure 3g. The excited‐state electrons become more easily to cross the energy barrier and reach the intersection of ^4^T_2_ and ^4^A_2_ energy levels as the temperature increases, leading to an increase in non‐radiative transition probability and the PL intensity of ALMSO:Cr^3+^ (A = Ca, Sr). Small Stokes shift also contributes to better thermal stability of the phosphor, and thus the CLMSO:Cr^3+^ sample has a more excellent thermal stability than SLMSO:Cr^3+^ because of the smaller Stokes shift of CLMSO:Cr^3+^ than SLMSO:Cr^3+^ (Table S7, Supporting Information). Based on the above analysis, we suspect that the superior thermal stability of ALMSO:Cr^3+^ (A = Ca, Sr) phosphors may be derived from the synergistic effect of Small Stokes shift, large bandgap, high energy barrier, robust structural rigidity, and relatively weak EPC effect.

Quantum efficiency (QE) is an important parameter to assess the potential commercial application of the phosphor. According to the energy gap law, NIR phosphors with emission wavelengths >800 nm usually suffer from poor thermal resistance and severe non‐radiative relaxation,^[^ ^60^, ^61^ ^]^ making the development of broadband NIR phosphors with both high quantum efficiency and excellent thermal stability remains a great challenge. As presented in Figure 3h and Figure S18, Supporting Information, the internal QE (IQE) of the optimized ALMSO:Cr^3+^ (A = Ca, Sr) samples was determined to be 82.5% and 78.8%, respectively. Owing to the relatively high absorption efficiency of the samples, the external QE (EQE) of ALMSO:Cr^3+^ (A = Ca, Sr) can be maintained at a comparatively high value (31.4% for CLMSO:Cr^3+^ and 34.3% for SLMSO:Cr^3+^). As mentioned above, a small Stokes shift allows more photons to go back to the ground state in the form of light emission, while the large bandgap can reduce the possibility of excited electrons being captured by the conduction band and facilitate the return of excited electrons to the ground state through irradiative transition. In consequence, the as‐prepared NIR‐emitting ALMSO:Cr^3+^ (A = Ca, Sr) phosphors feature remarkable quantum efficiency. To reflect the thermal stability and quantum efficiency performance of ALMSO:Cr^3+^ (A = Ca, Sr) samples, many developed Cr^3+^‐activated NIR phosphors with a peak wavelength over 800 nm are listed in Table S8 (Supporting Information) for comparison. As shown in Figure 3i, the quantum efficiency and thermal stability of our designed ALMSO:Cr^3+^ (A = Ca, Sr) NIR phosphors are superior to other reported NIR phosphors, including Na_2_CaHf_2_Ge_3_O_12_:Cr^3+^ (79.2%, 71.2%@423 K), Ga_4_GeO_8_:Cr^3+^ (60%, 56%@423 K), K_4_Ga_3_Ta(PO_4_)_6_:Cr^3+^ (27.4%, 35%@423 K), and so on.^[^ ^48^, ^62^, ^63^ ^]^

### 2.4 Photoluminescence Enhancement and Emission Spectrum Broaden

**Figure 4:**
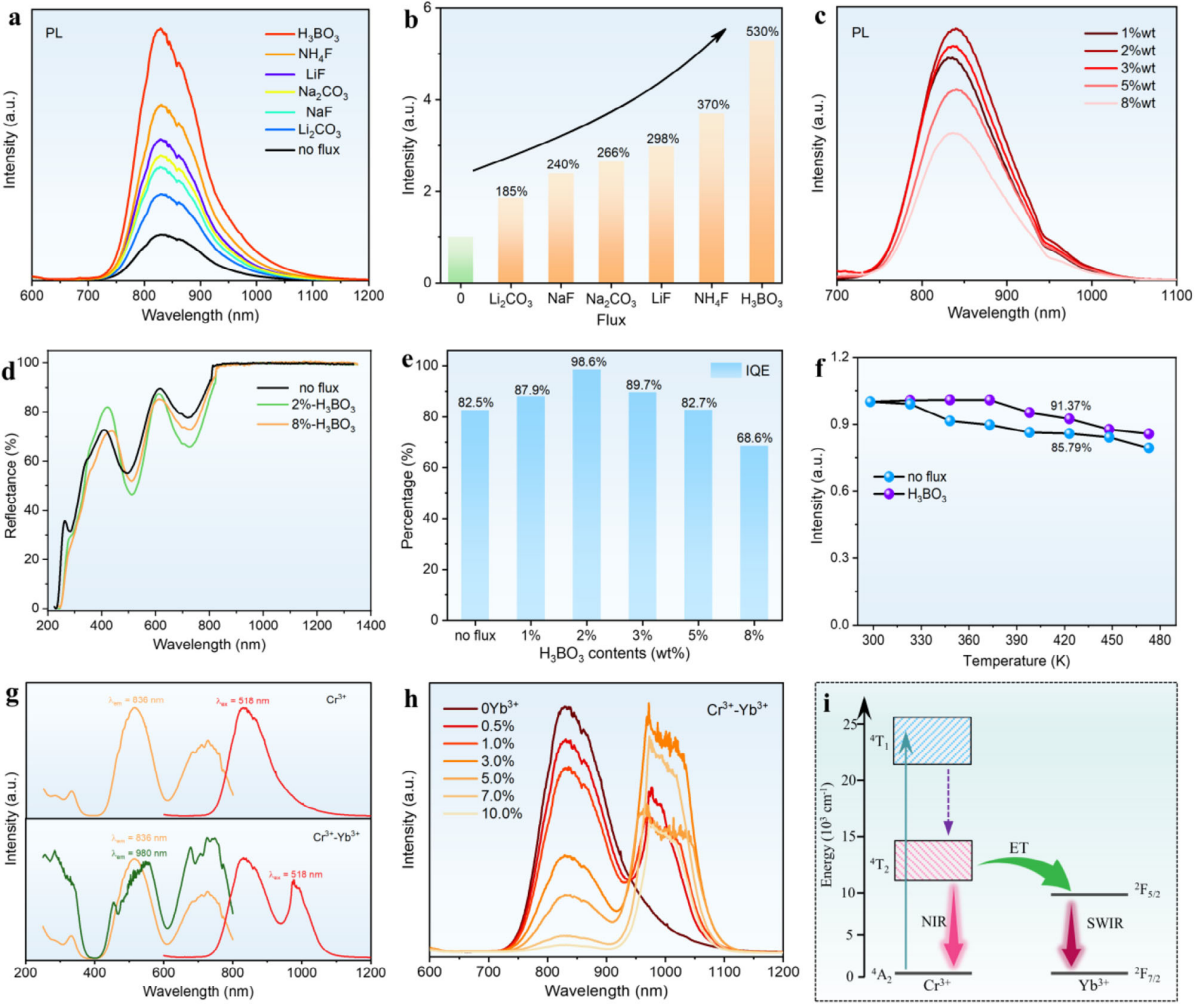
a) PL spectra, and b) Relative PL intensity of CLMSO:Cr^3+^ after adding different fluxes. c) PL spectra of CLMSO:Cr^3+^, y wt% H_3_BO_3_. d) DR spectra of CLMSO:Cr^3+^ sample with and without H_3_BO_3_ flux. e) H_3_BO_3_ content‐dependent IQE values in CMLSO:Cr^3+^. f) Temperature‐dependent PL intensity of CLMSO:Cr^3+^ and CLMSO:Cr^3+^, 2 wt% H_3_BO_3_ samples. g) PLE and PL spectra of CLMSO:Cr^3+^ before and after the introduction of Yb^3+^ ions. h) PL spectra of CLMSO:Cr^3+^, zYb^3+^ (z ═ 0–10.0%) samples. i) Schematic diagram for the Cr^3+^ → Yb^3+^ energy transfer process.

Flux strategy is one of the efficient approaches for enhancing the optical efficiency of the phosphors. Here, some typical fluxes were selected and introduced during the preparation to optimize the PL performance of CLMSO:Cr^3+^ in our study. As depicted in **Figure** **4**a,b and S19 (Supporting Information), all the added flux can contribute to the enhancement of Cr^3+^ NIR emission without changing the shape of the PLE and PL spectra. The strongest PL intensity is achieved with the addition of the H_3_BO_3_ flux, which exhibits over 530% higher NIR emission than that of the flux‐free sample. Among these fluxes to boost the NIR emission of CLMSO:Cr^3+^, the B^3+^ with a smaller ion radius can more easily promote its incorporation into the host lattice, which can compensate for the defects and thus enhance the luminescence intensity.^[^ ^64^ ^]^ Besides, the particle size of the sample has increased dramatically due to the addition of H_3_BO_3_ flux, which suggests that the improvement of the crystallinity of CLMSO:Cr^3+^ is also one of the reasons for the enhancement of the corresponding NIR emission (Figure S20, Supporting Information).

The effect of the additional amount of H_3_BO_3_ on the PL intensity of CLMSO:Cr^3+^ was further explored. The PL intensity of CLMSO:Cr^3+^, y wt% H_3_BO_3_ increases continuously and then decreases gradually, reaching the maximum at y ═ 2 wt% (Figure 4c). The DR spectra of CLMSO:Cr^3+^ sample with and without H_3_BO_3_ flux are displayed in Figure 4d. Although the absorption intensity of CLMSO:Cr^3+^, y wt% H_3_BO_3_ increases with the addition of H_3_BO_3_, the absorption peaks in the spectral range of 250–1350 nm are almost identical, demonstrating that no additional absorption center was introduced. The peak position of the DR and PL spectra is redshifted to a longer wavelength within several nanometers (Figure S21, Supporting Information), implying that the introduction of H_3_BO_3_ as the flux has a slight influence on the crystal field environment of CLMSO:Cr^3+^ phosphor. Furthermore, the quantum efficiency of CLMSO:Cr^3+^ with different H_3_BO_3_ contents was analyzed. The correlative measurement spectra are given in Figure S22 (Supporting Information). It shows that the IQE of CLMSO:Cr^3+^ can be further increased to 98.6% with the addition of H_3_BO_3_ content of 2 wt%, revealing that the introduction of H_3_BO_3_ flux agent facilitates the radiative recombination of Cr^3+^ ions (Figure 4e; Table S9, Supporting Information). In addition, the PL spectra for the two typical CLMSO:Cr^3+^ samples with and without the addition of H_3_BO_3_ flux at different temperatures were measured (Figure S23, Supporting Information). The normalized integrated NIR emission intensities vary with temperatures from 298 to 483 K are plotted in Figure 4f. The addition of H_3_BO_3_ flux incorporated into the host matrix contributes to the enhancement of crystallinity and benefits the improved thermal resistance properties. As a consequence, the thermal stability of CLMSO:Cr^3+^ is further increased from 85.79%@423 K to 91.37%@423 K after adding the optimal H_3_BO_3_ flux content. In short, the introduction of H_3_BO_3_ flux contributed to the improvement of luminescence efficiency and thermal quenching resistance performance simultaneously, making the CLMSO:Cr^3+^ phosphor suitable as a luminescence conversion material for practical NIR spectroscopy applications.

Although CLMSO:Cr^3+^ exhibits broadband emission in the range of 700–1200 nm, the emission intensity over 950 nm is still not high enough. To broaden the NIR emission spectrum, Yb^3+^ ion was introduced in CLMSO:Cr^3+^ phosphor as an extra NIR emitter to make up for the spectral deficiency beyond 950 nm. The absorption of Yb^3+^ and the emission of Cr^3+^ in CLMSO has considerable spectral overlap in the NIR region, verifying that the long‐wavelength emission of CLMSO:Cr^3+^ can be enhanced by constructing Cr^3+^ → Yb^3+^ energy transfer (ET) channel (Figure S24, Supporting Information). Figure 4g gives the PLE and PL spectra of CLMSO:Cr^3+^ and CLMSO:Cr^3+^, Yb^3+^ samples, respectively. The PLE spectra obtained by monitoring the emission at 836 nm (Cr^3+^) and 980 nm (Yb^3+^) are basically consistent with the single‐doped sample with Cr^3+^ ions, indicating the occurrence of an effective ET process from Cr^3+^ to Yb^3+^ because Yb^3+^ has a small absorption cross‐section and cannot be excited by visible light.^[^ ^65^ ^]^ After the incorporation of Yb^3+^ into the CLMSO:Cr^3+^ phosphor, a strong emission appeared at ≈1000 nm can be detected in addition to the characteristic broadband emission of the Cr^3+^ ion, which is associated with the ^2^F_5/2_ → ^2^F_7/2_ transition of Yb^3+^.^[^ ^66^ ^]^

The PL spectra of CLMSO:Cr^3+^, zYb^3+^ (z = 0–10.0%) with variation of Yb^3+^ concentrations upon 520 nm excitation are presented in Figure 4h. With the increase of Yb^3+^ content, the emission intensity of Yb^3+^ first significantly grows and then gradually declines, accompanied by the decrement of Cr^3+^ emission, further confirming the presence of the ET process between Cr^3+^ and Yb^3+^. PL decay studies demonstrated significantly shortened luminescent lifetimes of Cr^3+^ from 74.13 to 0.49 µs with an increase in Yb^3+^ doping concentration from 0% to 10%, and the calculated ET efficiency exhibits a continuous increase, reaching a peak value of nearly 100% (99.33%) when the Yb^3+^ doping concentration is 10.0% (Equations S5 and Figure S25, Supporting Information).^[^ ^17^ ^]^ Figure 4i depicts a simplified schematic diagram to describe the ET process from Cr^3+^ to Yb^3+^ ions. Under green light excitation, the electrons of Cr^3+^ ions are pumped from the ^4^A_2_ ground state to the ^4^T_1_ excited state and subsequently relaxed to the lowest ^4^T_2_ energy level through a non‐radiative transition. Some of the excited electrons return to the ground state ^4^A_2_, giving rise to the generation of Cr^3+^‐related NIR emission that covers from 700 to 1200 nm. Part of the excitation energy is transferred to the ^2^F_5/2_ energy level of Yb^3+^ when co‐doped with Yb^3+^ ion, resulting in long‐wavelength emission at ≈1000 nm through ^2^F_5/2_ → ^2^F_7/2_ transition. The influence of Yb^3+^‐doped on the crystal structure, luminescence efficiency, and thermal stability of CLMSO:Cr^3+^ phosphor is presented in Figure S26, Supporting Information. The pure monoclinic CLMSO phase can be well maintained after the incorporation of Yb^3+^ ions. With increasing Yb^3+^ contents, the IQE of CLMSO:Cr^3+^, zYb^3+^ (z = 0–10.0%) gradually decreases. The decreased IQE at higher Yb^3+^ concentrations may be caused by increased nonradiative transitions. Temperature‐dependent PL spectra of CLMSO:Cr^3+^ and CLMSO:Cr^3+^, Yb^3+^ are shown in Figure S26d,e (Supporting Information). The corresponding integrated intensities as a function of temperature revealed that 106.98% of the overall intensity can remain at 423 K for CLMSO:Cr^3+^, 1.0%Yb^3+^ (89.80%@423 K for CLMSO:Cr^3+^). It can be found that the Yb^3+^ emission (900‐1100 nm) shows reduced thermal quenching than the Cr^3+^ emission (700–900 nm), leading to enhanced thermal stability of Cr^3+^‐Yb^3+^ doped CLMSO phosphors.

### 2.5 Light Conversion Performance and NIR Spectroscopy Applications

**Figure 5:**
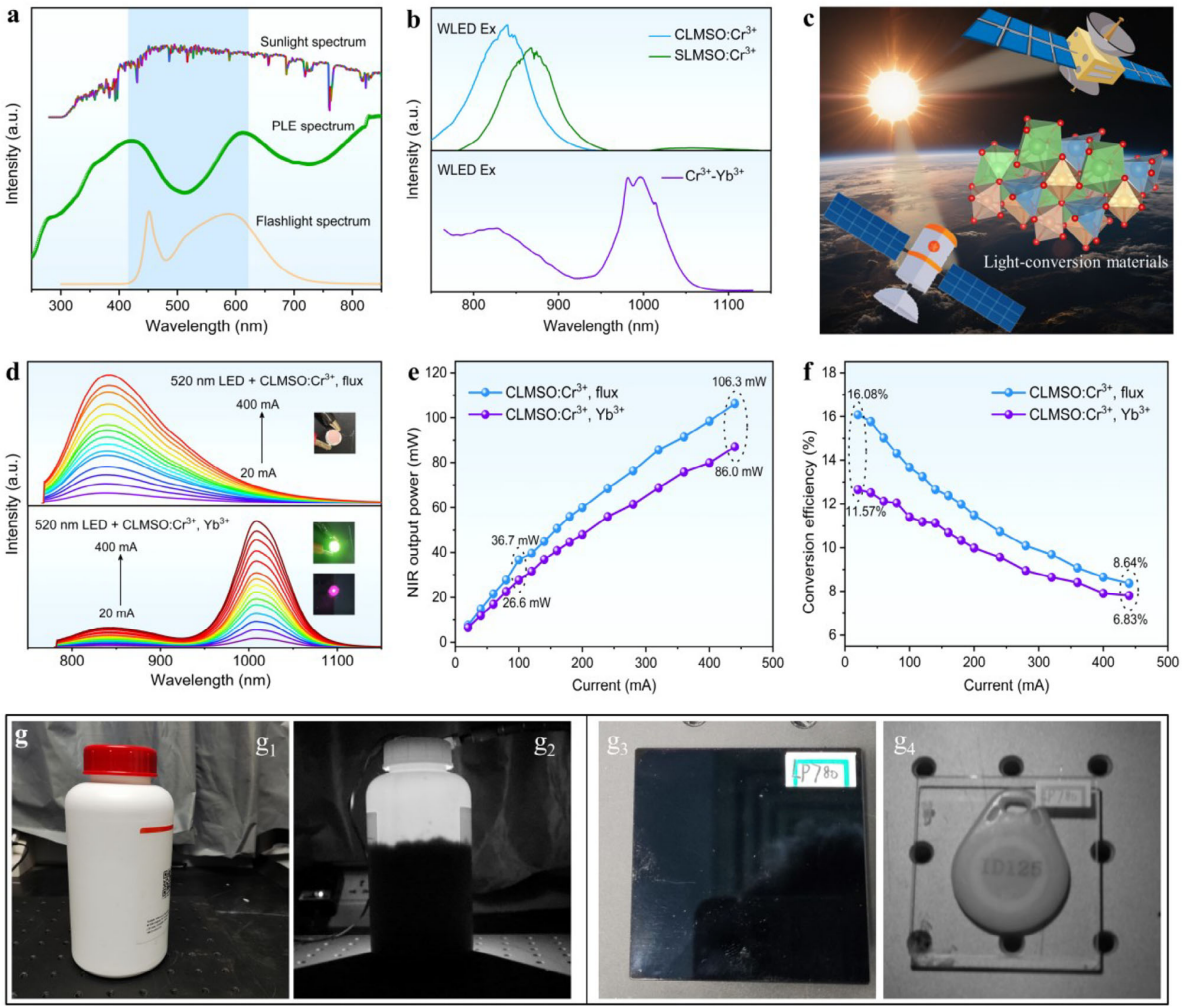
a) Comparison of PLE spectra of ALMSO:Cr^3+^, Yb^3+^ (A = Ca, Sr) phosphors with the spectra of smartphone flashlight and solar irradiation. b) Detected PL spectra of ALMSO:Cr^3+^, Yb^3+^ (A = Ca, Sr) pumped by white light LED. c) Schematic diagram of ALMSO:Cr^3+^, Yb^3+^ (A = Ca, Sr) phosphors for solar energy harvesting in outer space. d) EL spectra, e) NIR output power, and f) Photoelectronic conversion efficiency of the constructed NIR pc‐LED devices based on CLMSO:Cr^3+^, H_3_BO_3_ and CLMSO:Cr^3+^, Yb^3+^ samples at different driven currents. g) Demonstrated applications of the fabricated NIR pc‐LED devices for night vision and non‐destructive visualization.

Apart from the fascinating optical properties, the as‐prepared NIR phosphors exhibit excellent environmental resistance to ensure the foundation for their versatile applications (Figures S27, S28, Supporting Information). In addition, the designed NIR phosphors (taking CLMSO:Cr^3+^ as an example) demonstrate a full visible‐spectrum conversion ability that can be effectively excited by almost all visible light from 400 to 800 nm (Figure S29, Supporting Information). Notably, the solar irradiation and flashlight spectra exhibit an extremely large overlap region with the excitation spectra of the photonic materials (**Figure** **5**a). This spectral overlap gives us such an inspiration that whether our developed NIR phosphors can be pumped by natural sunlight or white light? To verify this hypothesis, a smartphone flashlight was used as the white light source and collected the optical signal using a homemade photo‐counting system. It can be found that intense NIR emission of ALMSO:Cr^3+^ (A = Ca, Sr) was successfully detected under white light pumping. Also, the Cr^3+^‐Yb^3+^ co‐doped samples can be effectively excited by white light, resulting in the corresponding characteristic emissions of Cr^3+^ and Yb^3+^ ions (Figure 5b) that match well with the high spectral response region of c‐Si solar cells.^[^ ^67^ ^]^ The emission intensity excited by white light LED is slightly higher than the sample pumped by green LED, which might be attributed to the fact that the spectral range of white light LED is broader than that of green LED (Figure S30, Supporting Information). Therefore, ALMSO:Cr^3+^, Yb^3+^ (A = Ca, Sr) phosphors are highly promising as light conversion materials for solar energy harvesting when integrated with photovoltaic cells, particularly when the rich solar irradiation in outer space is considered (Figure 5c).

Prototype NIR pc‐LED devices were constructed, combined with ALMSO:Cr^3+^, Yb^3+^ (A = Ca, Sr) phosphors and 520 nm LED chips to further estimate their potential NIR spectroscopy applications. When the driven current is on, bright NIR light emission can be observed using a 670 nm long‐pass filter. With the rise of operating currents from 20 to 400 mA, the emission intensities are monotonously increasing but do not yet reach saturation (Figure 5d; Figure S31, Supporting Information). Figure 5e,f presents the NIR output power and photoelectric conversion efficiency of the fabricated NIR pc‐LEDs at different driven currents. As the driven current increases from 20 to 400 mA, the NIR output power of CLMSO:Cr^3+^, H_3_BO_3_ and CLMSO:Cr^3+^, Yb^3+^ can reach 106.3 and 86.0 mW, respectively. At the working current of 20 mA, the corresponding photoelectric efficiency of CLMSO:Cr^3+^, H_3_BO_3_ and CLMSO:Cr^3+^, Yb^3+^ samples is calculated to be 16.08% and 11.57%, respectively. This indicates that the constructed NIR pc‐LEDs perform well in the aspect of output power and photoelectric conversion efficiency, surpassing most reported results on Cr^3+^‐activated NIR phosphors with emission wavelength over 800 nm under the same operating condition (Table S10, Supporting Information). When the ALMSO:Cr^3+^, Yb^3+^ (A = Ca, Sr) phosphors were encapsulated with 460 nm blue LED chips, excellent optoelectronic performance can also be obtained (Figures S32, S33, Supporting Information). As shown in Figure 5g, we can only see a white opaque plastic bottle and black optical filter by our naked eye or under natural visible light, while the concealed residual content of the drug and the ID number of the access card can be clearly observed with the help of NIR light from the constructed LED device, demonstrating the great promise of ALMSO:Cr^3+^, Yb^3+^ (A = Ca, Sr) phosphors for night vision and non‐destructive visualization. Therefore, utilizing the fabricated NIR LED device as a light source, bio‐friendly NIR light with powerful penetration ability may be able to replace X‐ray for dental analysis and identify the extent of tooth decay as NIR pc‐LEDs develop and mature in the future (Figures S34, Supporting Information).

## 3 Conclusion

In summary, a series of highly efficient ALMSO:Cr^3+^ (A = Ca, Sr) NIR phosphors with emission peaks over 830 nm has been successfully designed (λ_ex_ = 520 nm). The green light was utilized as an excitation source for the designed NIR phosphors to effectively reduce blue light hazards. Stokes shift is reduced in these Cr^3+^‐activated double perovskite phosphors through the cation substitution of A‐B sites. The broadband NIR emission is contributed to the simultaneous substitution of [SbO_6_] and [MgO_6_] polyhedral sites by Cr^3+^ ions, which is verified by Rietveld structural refinement, doping concentration‐dependent EPR spectra, TRPL spectra, Gaussian fitting of PL spectra, site occupation experiment, and DFT calculation. Benefiting from the small Stokes shift and relatively large bandgap to restrain non‐radiative transition and thermal ionization, ALMSO:Cr^3+^ (A = Ca, Sr) phosphors possess excellent thermal quenching resistance (89.80%@423 K) and high quantum efficiency (82.5%) simultaneously. Additionally, the PL intensity and emission spectra of ALMSO:Cr^3+^ (A = Ca, Sr) are effectively enhanced and broadened by the flux strategy and co‐doped with Yb^3+^ ions. The as‐prepared phosphors exhibited a full visible‐spectrum conversion ability from 400 to 800 nm that can be easily pumped by flashlight and natural sunlight, making ALMSO:Cr^3+^, Yb^3+^ (A = Ca, Sr) samples highly promising as light conversion materials for solar energy harvesting. The constructed prototype NIR pc‐LED device obtained a favorable NIR output power of 106.3 mW@400 mA and photoelectric conversion efficiency of 16.08%@20 mA, showing great potential in versatile NIR spectroscopy applications for night vision, non‐destructive visualization, and dental analysis. This work provides a new perspective on the development of long‐wavelength NIR phosphors with high luminescence efficiency and robust thermal stability for next‐generation portable NIR light sources.

## 4 Experimental Section

The detailed Experimental Section is provided in the Supporting Information file.

## Conflict of Interest

The authors declare no conflict of interest.

## Supporting information



Supporting Information

## Data Availability

The data that support the findings of this study are available from the corresponding author upon reasonable request.
