# Supplementary material for: Small Stokes Shift Induced Highly Efficient and Thermally Stable Broadband Near‐Infrared Antimonite Double Perovskite Emitters for Spectroscopy Applications
Source: Adv Sci (Weinh). 2025 Jul 12;12(38):e09583. doi: 10.1002/advs.202509583 (PMC12520467; doi:10.1002/advs.202509583)
Supplement: Supplementary file 1 — Supporting Information [file ADVS-12-e09583-s001.docx]

Supporting Information

Small Stokes Shift Induced Highly Efficient and Thermally Stable Broadband Near-Infrared Antimonite Double Perovskite Emitters for Spectroscopy Applications

Zhihao Zhou, Hongjun Jiang, Bozhao Yin, Guocheng Ji, Enhai Song, Jianrong Qiu, Zhongmin Yang, Guoping Dong*

**1. Experimental**

*Materials and Synthesis*: High-temperature solid-state reaction was used to prepare the Cr^3+^-doped ALaMgXO_6_ (A = Ca, Sr; X = Nb, Ta, Sb) double perovskite phosphors. CaCO_3_ (99.95%), SrCO_3_ (99.95%), La_2_O_3_ (99.99%), MgO (99%), Sb_2_O_3_ (99.95%), and Cr_2_O_3_ (99.95%) purchased from Aladdin were utilized as raw materials and weighted accurately according to the stoichiometric ratio. All raw materials were mixed by adding appropriate ethanol and ground thoroughly in an agate mortar for 10 min. After that, the mixtures were transferred into alumina crucibles and then calcined at 1773 K for 6 h in air atmosphere. Finally, the generated samples were cooled down to ambient temperature and reground into fine powers for subsequent analysis. Noted that CLMSO:Cr^3+^ phosphor with different types of fluxes (2 wt%) and Yb^3+^ doping concentrations (0-10.0%) were prepared by the same procedure.

*Measurements and Characterization*: X-ray diffraction (XRD) patterns of the samples were identified by an X-ray diffractometer (PANalytical Co., Netherlands). The GSAS software was used to conduct XRD Rietveld refinements. Electron paramagnetic resonance (EPR) spectra were collected by Bruker A300 spectrometer using the X-band frequency, and X-ray photoelectron spectroscopy (XPS) measurements were characterized by a Kratos Axis Ultra DLD spectrometer with Al Ka X-ray beam as the radiation source. Surface morphology, microstructure, elemental composition and distribution of the samples were analyzed using JEOL JEM-2100F transmission electron microscope (TEM) and Hitachi SU8600 scanning electron microscopy (SEM) equipped with an energy dispersive spectrometer (EDS). Thermoluminescence (TL) curves were collected with an FJ427A1 TL dosimeter. Diffuse reflection (DR) spectra were performed with a Lambda 950 UV-Vis-NIR spectrophotometer and used BaSO_4_ as standard reference. Photoluminescence (PL) and excitation (PLE) spectra (λ_ex_ = 520 nm), time-resolved PL (TRPL) spectra, and temperature-dependent PL spectra were recorded on an FLS920 fluorescence spectrometer (Edinburgh, UK). The excitation and emission slit width was set as 6 and 6, and NIR photomultiplier tube detector was used in the spectroscopy measurements. PL quantum efficiency of the as-prepared phosphors (λ_ex_ = 520 nm) was obtained utilizing a Vis-NIR absolute quantum efficiency test system (Hamamatsu C13534-11). To evaluate the formation energy and Debye temperature of Cr^3+^-doped ALaMgSbO_6_ (A = Ca, Sr) samples, density functional theory method was used based on MedeA-VASP package.

*LED device fabrication and application*: NIR pc-LED devices were fabricated by mixing ALaMgSbO_6_:Cr^3+^ (A = Ca, Sr) NIR phosphors with silicone adhesive thoroughly and then coated on 520 nm or 460 nm LED chips. Electroluminescence (EL) spectra and NIR output power of the manufactured NIR pc-LED devices were acquired by EVERFINE photoelectric measuring system (780-1650 nm). The demonstrated images for NIR pc-LED applications were captured through visible and NIR cameras, respectively.

**2. Equations**

$D_{\text{r}} =\frac{\text{|}R_{s}-R_{D}\text{|}}{R_{s}}\times100\%$ (S1)

where *R_S_* and *R_D_* represent the radius of the substituted ions and doped ions, respectively. The ionic radius difference between Sb^5+^/Mg^2+^ and Cr^3+^ ions in octahedral sites are 2.5% and 14.58%, which is lower than the threshold value 15% of good solubility. Therefore, it is supposed that both octahedral Sb and Mg sites can be occupied by the doped Cr^3+^ ions in theory.

$E_{form} =E\text{(}doped\text{)}-E\text{(}pure\text{)}-m\times\mu\text{(}dopant\text{)}+n\times\mu\text{(}atom\text{)}$ (S2)

where *E*(doped) and *E*(pure) represent the total energies of the doped and un-doped system, *m* and *n* as well as *μ*(dopant) and *μ*(atom) are the atomic potential energy and the number of dopants and substituted atoms, respectively.

 (S3)

$f\text{(}\upsilon\text{)}=\left\{3\left[2\left(\frac{2}{3}\cdot\frac{1+\upsilon}{1-2\upsilon}\right)^{3/2}+\left(\frac{1}{3}\cdot\frac{1+\upsilon}{1-\upsilon}\right)^{3/2}\right]^{-1}\right\}^{1/3}$ (S4)

where *ħ* and *k* respectively represent the simplified Planck constant and Boltzmann constant, *B_H_* denotes the bulk modulus of the crystal, *v* is the Poisson ratio, and *V*, *N*, and *M* are the volume, number of atoms, and molecular mass of the unit cell, respectively.

The energy transfer efficiency (η_ET_) is calculated by the following equation:

$\eta_{\text{ET}} =1-\frac{\tau}{\tau_{0}}$ (S5)

where *τ* and *τ_0_* are the lifetimes of Cr^3+^ in the presence and absence of Yb^3+^, respectively.

**3. Figures**





**Figure S1**. (a) Normalized PLE spectra, and (b) PLE and PL spectra of CaGdMgSbO_6_:Cr^3+^, CaLaMgSbO_6_:Cr^3+^, and SrLaMgSbO_6_:Cr^3+^ phosphors, respectively.





**Figure S2**. XRD patterns of (a) CLMSO:Cr^3+^, and (b) SLMSO:Cr^3+^ phosphors with different Cr^3+^ doping concentrations.





**Figure S3**. XRD Rietveld refinement of ALMSO (A = Ca, Sr) matrixes.


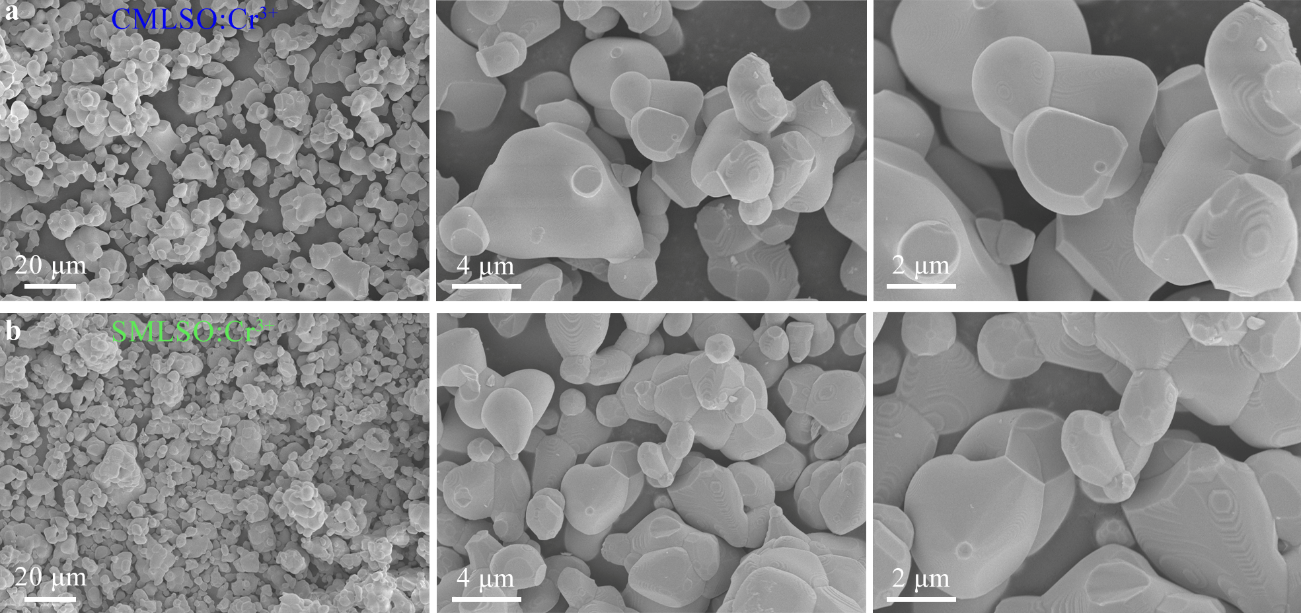


**Figure S4**. SEM images of ALMSO:Cr^3+^ (A = Ca, Sr) samples with different magnifications.


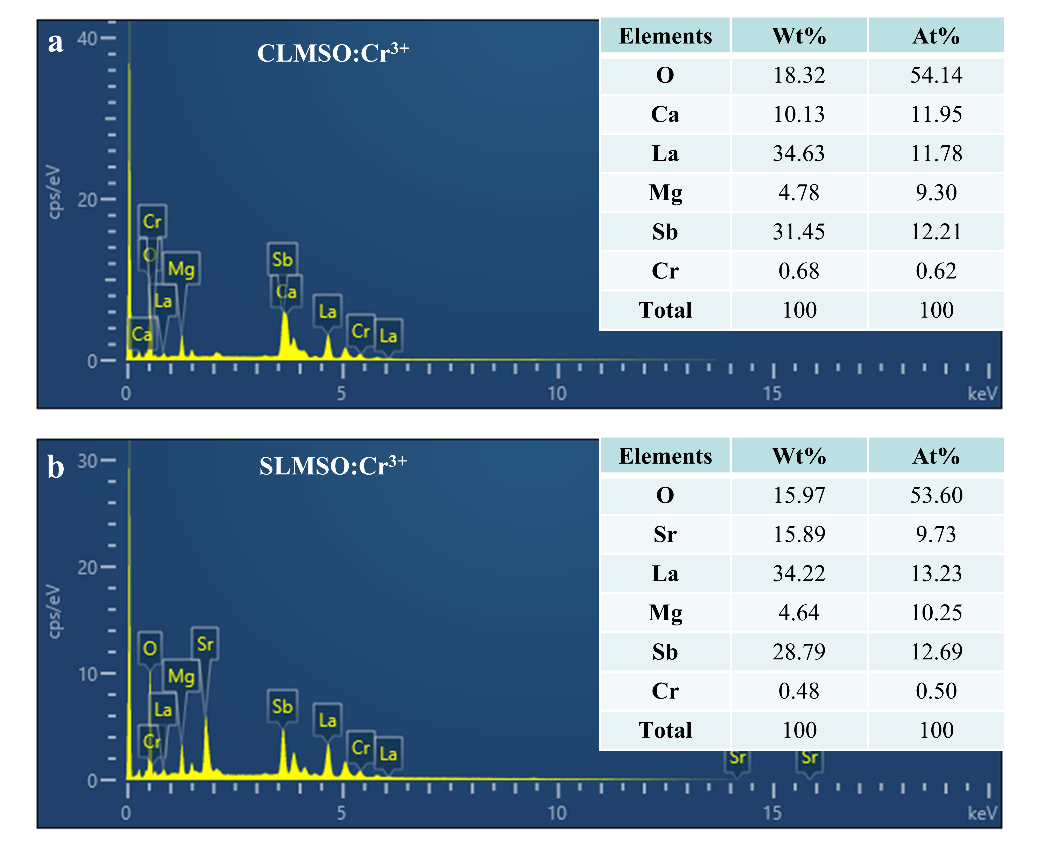


**Figure S5**. EDS spectrum of ALMSO:Cr^3+^ (A = Ca, Sr) samples.

EDS results demonstrate that the atomic ratio of the elements in ALMSO:Cr^3+^ (A = Ca, Sr) is close to the nominal stoichiometric ratio.





**Figure S6**. High-resolution Cr 2*p* XPS spectra of ALMSO:Cr^3+^ (A = Ca, Sr) samples.





**Figure S7**. (a, b) DR spectra of ALMSO (A = Ca, Sr) matrixes and Cr^3+^-doped samples. The inset shows the photographs of the matrixes and doped samples under natural light. (c, d) Calculated bandgap value of ALMSO (A = Ca, Sr) matrixes determined by the plot of [*F(R)hυ*]^2^ *versus* energy (eV).





**Figure S8**. (a, c) PLE, and (b, d) PL spectra of ALMSO:Cr^3+^ (A = Ca, Sr) with different Cr^3+^ doping concentrations.





**Figure S9**. Relation between Log (*I/x*) and Log (*x*) in ALMSO:Cr^3+^ (A = Ca, Sr).

The type of multipolar-multipolar interaction can be represented by the following formula:

$$\frac{1}{x}=\frac{k}{1+\beta(x)^{\theta/3}}$$

where *I* is the emission intensity, *k* and *β* are constants depending on the interaction type and the host lattice, *θ* is the index of electric multipole, and *x* represents the doping concentration.





**Figure S10**. (a, c) Normalized PLE, and (b, d) Normalized PL spectra of ALMSO:Cr^3+^ (A = Ca, Sr) with different Cr^3+^ doping concentrations.


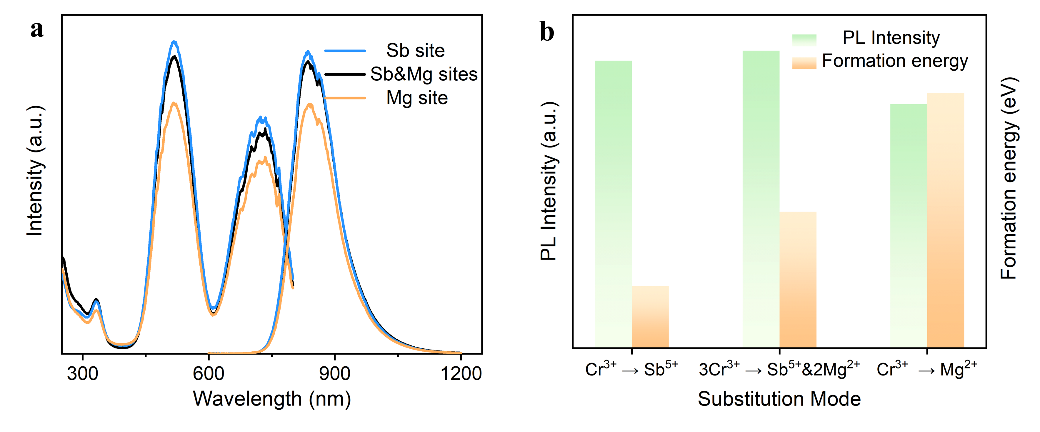


**Figure S11**. (a) PLE and PL spectra, (b) Corresponding PL intensity and formation energy analysis of Cr^3+^ doped at different substitution modes.





**Figure S12**. Gaussian fitting of the normalized PL spectra and the Cr1 and Cr2 ratio changes with Cr^3+^ concentrations in CLMSO:x%Cr^3+^ (x = 0.5-15).

When the doping concentration is low, Cr^3+^ ion will first choose to enter the Sb^5+^ site and thus the Cr1 center contributes more to PL than the Cr2 center. As the content of Cr^3+^ increases, the interaction between the Cr^3+^-Cr^3+^ pairs will be strengthened and thus Cr2 center contributes more to PL at higher doping concentrations.

**

**

**Figure S13**. Gaussian fitting of the normalized PL spectra and the Cr1 and Cr2 ratio changes with Cr^3+^ concentrations in SLMSO:x%Cr^3+^ (x = 0.5-15).

When the doping concentration is low, Cr^3+^ ion will first choose to enter the Sb^5+^ site and thus the Cr1 center contributes more to PL than the Cr2 center. As the content of Cr^3+^ increases, the interaction between the Cr^3+^-Cr^3+^ pairs will be strengthened and thus Cr2 center contributes more to PL at higher doping concentrations.





**Figure S14**. Temperature-dependent PL spectra of (a) CLMSO:Cr^3+^, and (b) SLMSO:Cr^3+^, respectively.

**

**

**Figure S15**. Cycling stability of CLMSO:Cr^3+^ phosphors in the range from RT to 423 K.

**
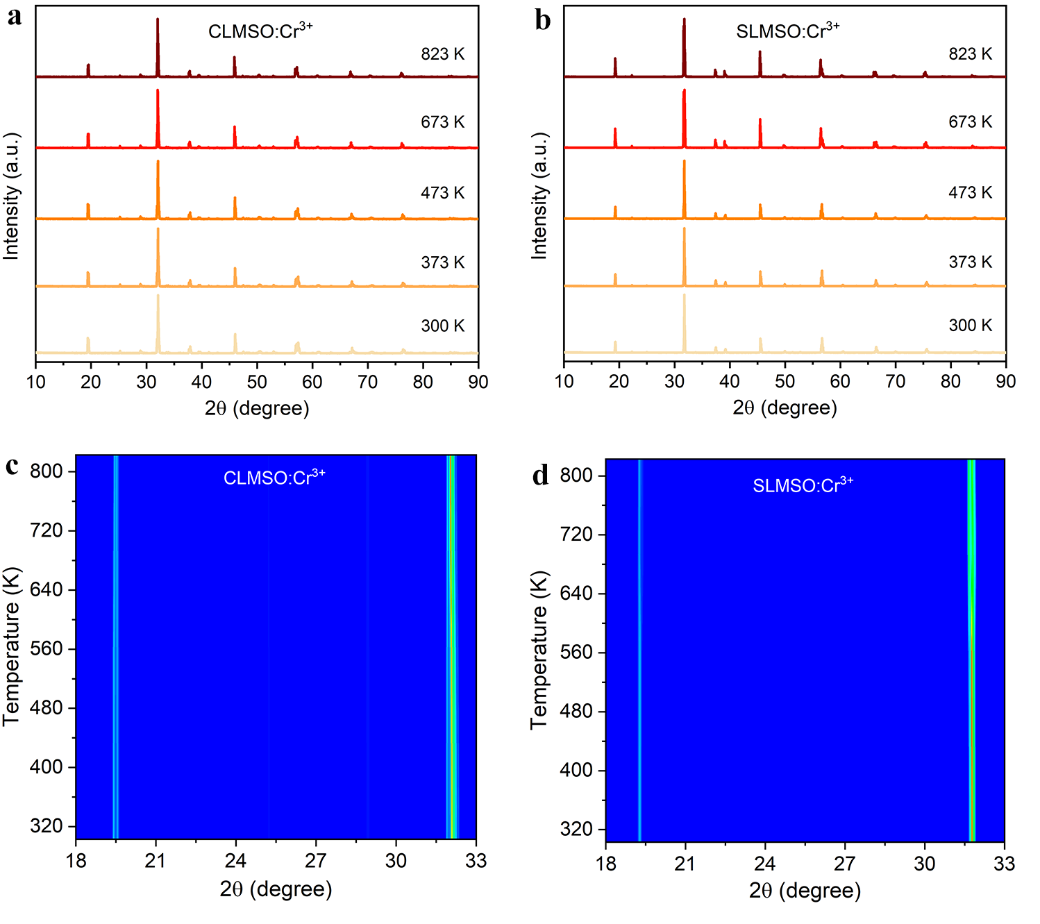
**

**Figure S16**. (a, b) *In situ* XRD patterns, and (c, d) Enlarged XRD patterns of ALMSO:Cr^3+^ (A = Ca, Sr) recorded at different temperatures.





**Figure S17**. TG and DSC curves of ALMSO:Cr^3+^ (A = Ca, Sr) samples.





**Figure S18**. Quantum efficiency measurement of ALMSO:Cr^3+^ (A = Ca, Sr) phosphors (λ_ex_ = 520 nm).





**Figure S19**. (a) PLE and (b) PL spectra of CLMSO:Cr^3+^ after adding different fluxes.

All the added flux can contribute to the enhancement of Cr^3+^ NIR emission without changing the shape of the PLE and PL spectra, demonstrating that no additional absorption center was introduced.


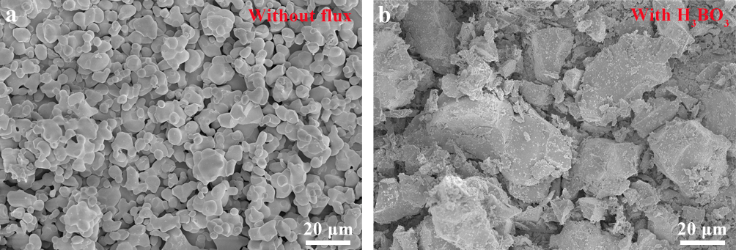


**Figure S20**. SEM images of CMLSO:Cr^3+^ samples before and after the introduction of H_3_BO_3_.





**Figure S21**. (a) PLE and (b) PL spectra of CLMSO:Cr^3+^ with variation of H_3_BO_3_ contents.





**Figure S22**. Quantum efficiency measurement of CLMSO:Cr^3+^ with different H_3_BO_3_ contents (λ_ex_ = 520 nm).





**Figure S23**. Temperature-dependent PL spectra of (a) CLMSO:Cr^3+^, and (b) CLMSO:Cr^3+^, H_3_BO_3_, respectively.





**Figure S24**. DR spectra of CLMSO:Cr^3+^ and CLMSO:Cr^3+^, Yb^3+^, and PL spectra of CLMSO:Cr^3+^.

The DR spectrum of CLMSO:Cr^3+^, Yb^3+^ exhibits an absorption band in the range of 850-1050 nm, which belongs to the ^2^F_7/2_ → ^2^F_5/2_ characteristic absorption of Yb^3+^ ions. In addition, the absorption of Yb^3+^ has a large overlap with the emission spectrum of CLMSO:Cr^3+^ sample, suggesting that energy transfer can be constructed in CLMSO:Cr^3+^.





**Figure S25**. (a) PL decay curves of CLMSO:Cr^3+^, zYb^3+^ (z = 0-10.0%) by monitoring the emission at 836 nm of Cr^3+^. (b) PL lifetimes and energy transfer efficiency as a function of Cr^3+^ doping concentrations.

PL decay studies demonstrated significantly shortened luminescent lifetimes of Cr^3+^ from 74.13 to 0.49 μs with an increase in Yb^3+^ doping concentration from 0% to 10%. Such an exceptionally pronounced change of Cr^3+^ luminescent lifetimes are mainly caused by the relatively high doping concentrations of Yb^3+^ ions (10.0%), and the excitation energy can be almost transferred to Yb^3+^ to generate strong emission appeared at around 1000 nm. The calculated ET efficiency exhibits a continuous increase, reaching a peak value of nearly 100% (99.33%) when the Yb^3+^ doping concentration is 10.0%.


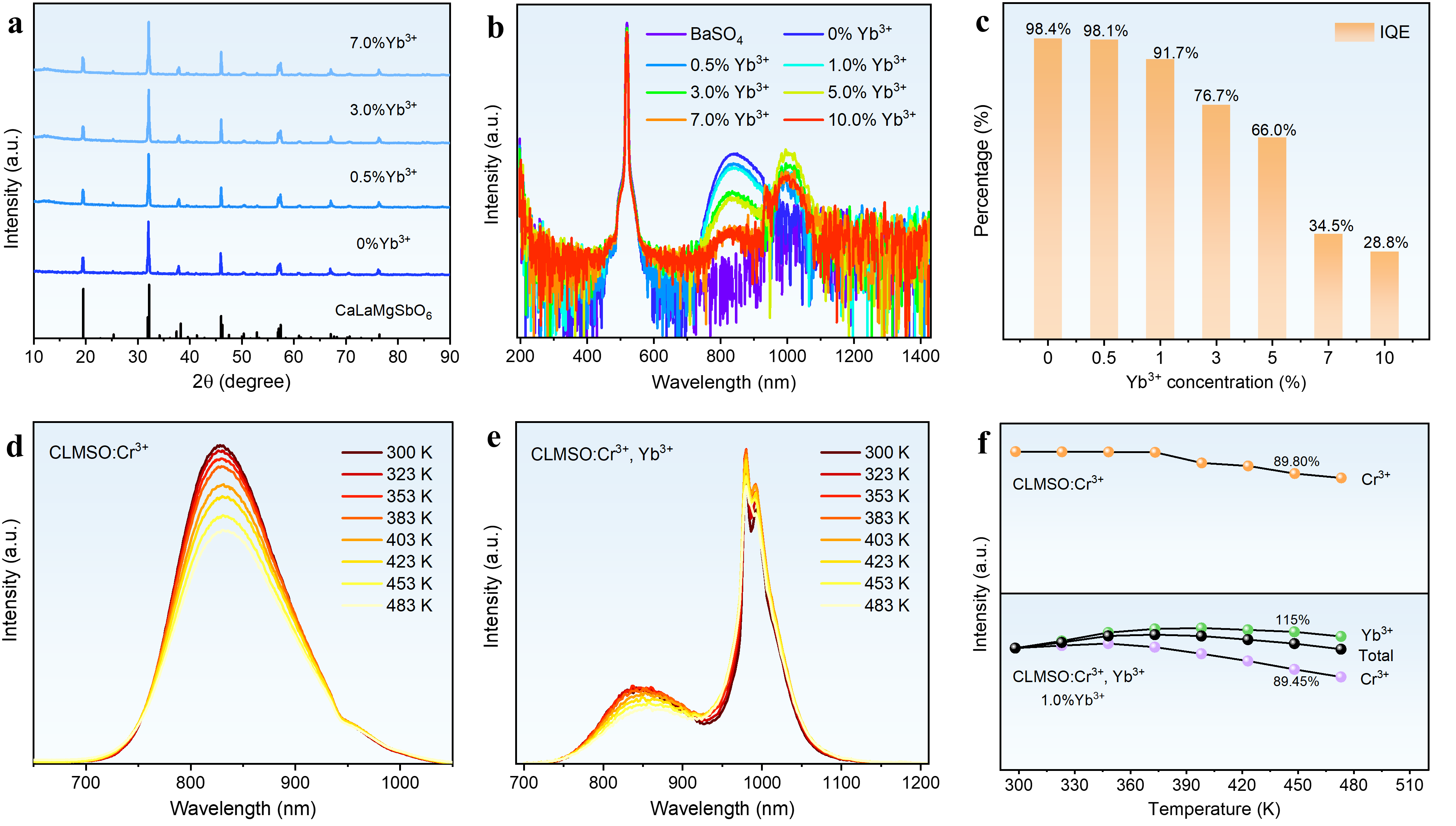


**Figure S26**. (a) XRD patterns, (b) Quantum efficiency measurement, and (c) IQE values of CLMSO:Cr^3+^ with different Yb^3+^ concentrations. (d-e) Temperature-dependent PL mapping, and (f) Integral PL intensity of CLMSO:Cr^3+^ and CLMSO:Cr^3+^, 1.0%Yb^3+^ phosphors.

The reduced thermal quenching is found with Yb^3+^ codoping. The enhanced thermal stability with Yb^3+^ codoping is mainly due to the energy transfer from Cr^3+^ to thermally more stable Yb^3+^. After the Yb^3+^ codoping, considerable excitation energy is transferred to Yb^3+^ ions. In this case, the energy loss *via* the thermally activated nonradiative transition of Cr^3+^ is suppressed and the thermally stable Yb^3+^ emission contributes to the total emissions. Thus, reduced thermal quenching is achieved for the total emissions of Cr^3+^-Yb^3+^ doped CLMSO phosphors.


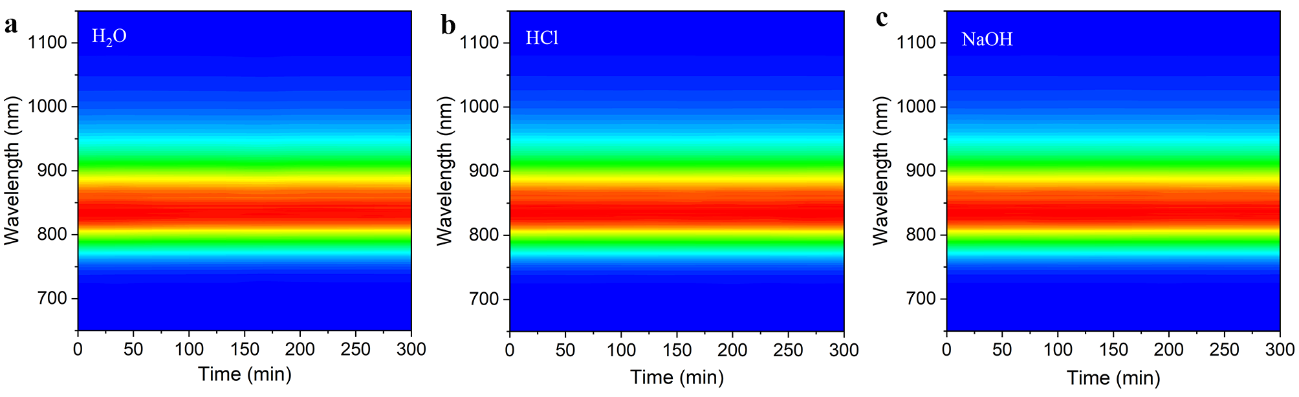


**Figure S27**. PL spectra mapping of CLMSO:Cr^3+^ phosphor after immersing in (a) water, (b) HCl solution, and (c) NaOH solution for 300 min.





**Figure S28**. (a) XRD patterns, and (b) PL spectra of CLMSO:Cr^3+^ before and after being stored in the air for one year.





**Figure S29**. Emission spectra mapping at different excitation wavelengths (𝜆_ex_ = 400-800 nm) of CLMSO:Cr^3+^.

The designed NIR phosphors (taking CLMSO:Cr^3+^ as an example) demonstrate a full visible-spectrum conversion ability that can be effectively excited by almost the entire visible light from 400 to 800 nm.





**Figure S30**. (a) Emission spectra of white light and green LED used as excitation source for CLMSO:Cr^3+^ and CLMSO:Cr^3+^, Yb^3+^ phosphors, PL spectra of (b) CLMSO:Cr^3+^ and (c) CLMSO:Cr^3+^, Yb^3+^ excited by white light and green LED, respectively (collected from Ocean Optics fiber optic spectrometer).

The emission intensity generated by the excitation of white light LED is slightly greater than that of the sample pumped by green LED. This might be attributed to the fact that the spectral range of white light LED is broader than that of green LED, which can more effectively excite the samples to produce NIR emission.





**Figure S31**. (a, c) EL spectra, and (b, d) NIR output power and photoelectric conversion efficiency of the fabricated NIR pc-LED devices (packing with 520 nm blue LED chip) based on ALMSO:Cr^3+^ (A = Ca, Sr) phosphors as the driven current increases from 20 to 400 mA.





**Figure S32**. EL spectra of the fabricated NIR pc-LED devices (packing with 460 nm blue LED chip) based on (a) CLMSO:Cr^3+^, (b) SLMSO:Cr^3+^, (c) CLMSO:Cr^3+^, H_3_BO_3_, and (d) CLMSO:Cr^3+^, Yb^3+^ phosphors.





**Figure S33**. NIR output power and photoelectric conversion efficiency of the fabricated NIR pc-LED devices (packing with 460 nm blue LED chip) based on (a) CLMSO:Cr^3+^, (b) SLMSO:Cr^3+^, (c) CLMSO:Cr^3+^, H_3_BO_3_, and (d) CLMSO:Cr^3+^, Yb^3+^ phosphors.


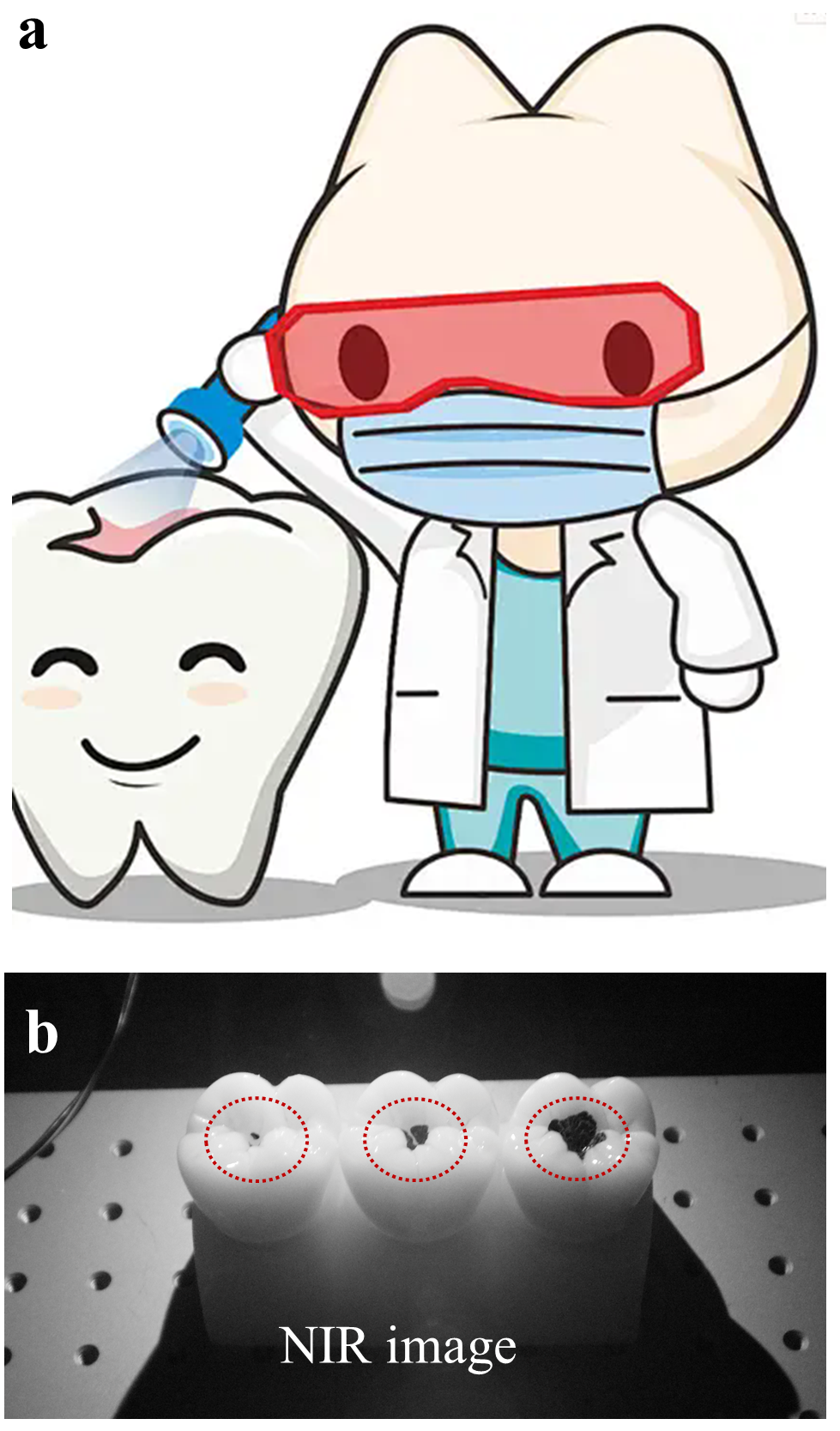


**Figure S34**. Potential application of ALMSO:Cr^3+^, Yb^3+^ (A = Ca, Sr) phosphors as a light source for dental analysis.

X-rays are often used to evaluate the surface of teeth (Figure S34a), but biological safety of X-rays become a new issue since long-term X-ray irradiation inevitably causes radiation damage to healthy tissues. As shown in Figure S34b, utilizing the fabricated NIR LED device as a light source, bio-friendly NIR light with powerful penetration ability can make it easy to see the extent of tooth decay and tiny structures within the oral cavity in the NIR range, which may provide a potential method (combine with optical fibers to transmit images) to replace X-ray for dental analysis as NIR pc-LEDs develop and mature in the future.

**4. Tables**

**Table S1.** Optical properties of previously reported double-perovskite NIR phosphors doped with Cr^3+^ ions.

| **NIR Phosphors** | **Emission peak (nm)** | **IQE (%)** | **PL intensty@423 K (%)** | **References** |
| --- | --- | --- | --- | --- |
| **Mg_2_InSbO_6_** | 717 | 70.8 | 60 | [1] |
| **Mg_2_LaTaO_6_** | 792 | 53.76 | 76.48 | [2] |
| **Ca_2_AlTaO_6_** | 741 | 45.07 | 94 | [3] |
| **Ca_2_AlNbO_6_** | 744 | 32.6 | 54 | [4] |
| **Ca_2_ScTaO_6_** | 830 | 32.8 | 60 | [5] |
| **Ca_2_ScNbO_6_** | 858 | — | 20.4 | [6] |
| **Ca_2_InTaO_6_** | 880 | 33.6 | ＜30 | [7] |
| **Ca_2_InNbO_6_** | 935 | 15.8 | 29.6 | [8] |
| **Sr_2_GaTaO_6_** | 740 | 77.1 | 40 | [9] |
| **Sr_2_AlNbO_6_** | 760 | 66.1 | 52 | [10] |
| **Sr_2_GaSbO_6_** | 885 | 14.2 | 60 | [11] |
| **Sr_2_ScSbO_6_** | 890 | 82 | 66.4 | [12] |
| **Sr_2_ScTaO_6_** | 948 | 69.8 | 54 | [13] |
| **Sr_2_InSbO_6_** | 920 | 63.2 | 37 | [11] |
| **SrLaMgTaO_6_** | 810 | 37.8 | 80.4 | [14] |
| **BaLaMgNbO_6_** | 830 | 50.9 | 20 | [15] |
| **NaLaMgWO_6_** | 820 | 16.7 | 20 | [16] |
| **LiYMgWO_6_** | 910 | 48.85 | ＜40 | [17] |
| **La_2_MgSnO_6_** | 751 | 6.3 | 68.5 | [18] |
| **La_2_MgHfO_6_** | 776 | 60 | 61.4 | [19] |
| **La_2_MgZrO_6_** | 825 | 58 | 53 | [20] |
| **La_2_CaZrO_6_** | 844 | 42.6 | ＜50 | [21] |
| **La_2_CaHfO_6_** | 906 | 8.2 | — | [22] |

**Table S2.** Unit cell parameters and Rietveld refinement results of un-doped and Cr^3+^-doped ALMSO (A = Ca, Sr) samples.

| **Samples** | **CLMSO host** | **CLMSO:Cr^3+^** | **SLMSO host** | **SLMSO:Cr^3+^** |
| --- | --- | --- | --- | --- |
| **space group** | *P2_1_/n* | *P2_1_/n* | *P2_1_/n* | *P2_1_/n* |
| **crystal system** | Monoclinic | Monoclinic | Monoclinic | Monoclinic |
| ***a* (Å)** | 5.5859 | 5.5867 | 5.6770 | 5.6807 |
| ***b* (Å)** | 5.7280 | 5.7290 | 5.7111 | 5.7137 |
| ***c* (Å)** | 9.6804 | 9.6809 | 9.8340 | 9.8359 |
| ***V* (Å^3^)** | 252.995 | 253.076 | 260.455 | 260.736 |
| ***Z*** | 2 | 2 | 2 | 2 |
| ***α*** | 90.000° | 90.000° | 90.000° | 90.100° |
| ***β*** | 125.232° | 125.236° | 125.245° | 125.225° |
| ***γ*** | 90.000° | 90.006° | 90.000° | 89.860° |
| ***R_wp_*** | 11.09% | 10.81% | 11.35% | 11.05% |
| **χ^2^** | 2.08 | 2.05 | 3.56 | 3.42 |

**Table S3.** Main Mg/Sb-O bond lengths and O-Mg/Sb-O bond angles of ALMSO (A = Ca, Sr) compounds.

| **Compounds** | **Mg-O bonds (Å)** | **Sb-O bonds (Å)** | **O-Mg-O bond angles** | **O-Sb-O bond angle** |
| --- | --- | --- | --- | --- |
| **CLMSO** | 2.10114 | 2.03925 | 89.3234° | 91.3593° |
|  | 2.16503 | 2.04437 | 91.4245° | 88.9540° |
|  | 2.13282 | 2.04686 | 92.6231° | 91.0221° |
|  | 2.16246 | 2.05951 | 88.6800° | 91.8224° |
|  | 2.09194 | 2.04792 | 92.5098° | 88.4411° |
|  | 2.12835 | 2.05167 | 92.0422° | 90.7283° |
| **Average** | **2.13029** | **2.04826** | **91.1005**° | **90.3878**° |
| **Distortion index** | ***Dis*_[MgO6]_ = 0.012** | | ***Dis*_[SbO6]_ = 0.003** | |
| **SLMSO** | 2.14288 | 2.04904 | 91.2537° | 93.4438° |
|  | 2.13798 | 2.04052 | 89.9798° | 89.0303° |
|  | 2.13399 | 2.05254 | 92.9413° | 90.6845° |
|  | 2.10853 | 2.04377 | 90.8911° | 89.1415° |
|  | 2.11638 | 2.05852 | 85.7236° | 89.9032° |
|  | 2.13796 | 2.04362 | 89.6474° | 91.1568° |
| **Average** | **2.12962** | **2.04800** | **90.0728**° | **90.5600**° |
| **Distortion index** | ***Dis*_[MgO6]_ = 0.006** | | ***Dis*_[SbO6]_ =0.002** | |

The distortion index (*D*_dis_) of [MgO_6_] and [SbO_6_] can be calculated by the following equation:

$$D_{\text{dis}}=\frac{1}{n}\sum_{i=1}^{n} \frac{\left|d_{i}-d_{av} \right|}{d_{av}}$$

where *D*_dis_ is the distortion index of the polyhedron, *n* is the coordination number of central ion, *d*_i_ is the distance of central ion to the *i*-th coordinating atom, and *d*_ave_ is their average bond length.

**Table S4.** Crystal field parameters of Cr^3+^ in CLMSO and SLMSO.

| **Samples** | **^4^A_2_ → ^4^T_1_ (cm^-1^)** | **^4^A_2_ → ^4^T_2_ (cm^-1^)** | **ΔS (cm^-1^)** | ***D_q_* (cm^-1^)** | ***B* (cm^-1^)** | ***D_q_/B*** |
| --- | --- | --- | --- | --- | --- | --- |
| CLMSO:Cr^3+^ | 19230.77 | 13586.95 | 1625.23 | 1277.43 | 586.34 | 2.18 |
| SLMSO:Cr^3+^ | 19011.41 | 13513.51 | 1966.17 | 1253.04 | 569.45 | 2.20 |

**Table S5.** Crystal field parameters of ALMSO:Cr^3+^ (A = Ca, Sr) samples with different Cr^3+^ concentrations.

| **Samples** | **Concentrations** | **^4^A_2_ → ^4^T_1_ (cm^-1^)** | **^4^A_2_ → ^4^T_2_ (cm^-1^)** | **ΔS (cm^-1^)** | ***D_q_* (cm^-1^)** | ***D_q_/B*** |
| --- | --- | --- | --- | --- | --- | --- |
| **CLMSO:Cr^3+^** | 0.5% | 19230.77 | 13586.95 | 1596.55 | 1278.87 | 2.182 |
|  | 1.0% | 19230.77 | 13586.95 | 1625.23 | 1277.43 | 2.179 |
|  | 2.0% | 19230.77 | 13586.95 | 1625.23 | 1277.43 | 2.179 |
|  | 3.0% | 19230.77 | 13586.95 | 1738.61 | 1271.76 | 2.165 |
|  | 5.0% | 19230.77 | 13586.95 | 1738.61 | 1271.76 | 2.165 |
|  | 7.0% | 19230.77 | 13586.95 | 1752.64 | 1271.06 | 2.163 |
|  | 10.0% | 19230.77 | 13586.95 | 1752.64 | 1271.06 | 2.163 |
|  | 12.0% | 19230.77 | 13586.95 | 1766.62 | 1270.36 | 2.161 |
|  | 15.0% | 19230.77 | 13586.95 | 1766.625 | 1270.36 | 2.161 |
| **SLMSO:Cr^3+^** | 0.5% | 19011.41 | 13513.51 | 1939.44 | 1254.38 | 2.204 |
|  | 1.0% | 19011.41 | 13513.51 | 1966.17 | 1253.04 | 2.200 |
|  | 2.0% | 19011.41 | 13513.51 | 1966.17 | 1253.04 | 2.200 |
|  | 3.0% | 19011.41 | 13513.51 | 1992.78 | 1251.71 | 2.197 |
|  | 5.0% | 19011.41 | 13513.51 | 1992.78 | 1251.71 | 2.197 |
|  | 7.0% | 19011.41 | 13513.51 | 2019.26 | 1250.39 | 2.194 |
|  | 10.0% | 19011.41 | 13513.51 | 2019.26 | 1250.39 | 2.194 |
|  | 12.0% | 19011.41 | 13513.51 | 2045.62 | 1249.07 | 2.190 |
|  | 15.0% | 19011.41 | 13513.51 | 2071.86 | 1247.76 | 2.187 |

**Table S6.** Fitting parameters of PL spectra of ALMSO:Cr^3+^ (A = Ca, Sr) samples with different Cr^3+^ concentrations.

| **Samples** | **Concentrations** | **Cr1 (Sb^5+^ octahedral sites)** | | | **Cr2 (Mg^2+^ octahedral sites)** | |  |
| --- | --- | --- | --- | --- | --- | --- | --- |
|  |  | **Position (cm^-1^)** | **Percentage** | | **Position (cm^-1^)** | **Percentage** | |
| **CLMSO:Cr^3+^** | 0.5% | 12000.41 | 73.48% | | 11020.93 | 26.52% | |
|  | 1.0% | 12000.72 | 70.68% | | 11020.34 | 29.32% | |
|  | 2.0% | 12000.72 | 69.45% | | 11020.93 | 30.55% | |
|  | 3.0% | 12000.83 | 65.46% | | 11020.50 | 34.54% | |
|  | 5.0% | 12000.48 | 64.68% | | 11020.15 | 35.32% | |
|  | 7.0% | 12000.48 | 63.07% | | 11020.15 | 36.93% | |
|  | 10.0% | 12000.48 | 56.24% | | 11020.15 | 43.76% | |
|  | 12.0% | 12000.48 | 55.40% | | 11020.15 | 44.60% | |
|  | 15.0% | 12000.48 | 51.92% | | 11020.15 | 48.08% | |
| **SLMSO:Cr^3+^** | 0.5% | 11734.48 | 82.00% | | 10640.15 | 18.00% | |
|  | 1.0% | 11734.48 | 81.88% | | 10640.15 | 18.12% | |
|  | 2.0% | 11734.48 | 80.98% | | 10640.15 | 19.02% | |
|  | 3.0% | 11734.48 | 74.12% | | 10640.15 | 25.88% | |
|  | 5.0% | 11734.48 | 71.53% | | 10640.15 | 28.47% | |
|  | 7.0% | 11734.48 | 65.88% | | 10644.15 | 34.12% | |
|  | 10.0% | 11730.48 | 60.72% | | 10640.15 | 39.28% | |
|  | 12.0% | 11734.48 | 55.89% | | 10640.15 | 44.11% | |
|  | 15.0% | 11734.48 | 55.47% | 10645.15 | | 44.53% | |

**Table S7.** Comparison of some typical parameters between CLMSO:Cr^3+^ and SLMSO:Cr^3+^ phosphors.

| **Samples** | **T_423 K_ (%)** | **IQE (%)** | **Bandgap (eV)** | **Debye temperature (K)** | **Stokes shift (cm^-1^)** |
| --- | --- | --- | --- | --- | --- |
| CLMSO:Cr^3+^ | 89.80 | 82.5 | 4.85 | 475.46 | 1625.24 |
| SLMSO:Cr^3+^ | 84.38 | 78.8 | 4.78 | 465.84 | 1966.17 |

**Table S8.** Optical properties of ALMSO:Cr^3+^ (A = Ca, Sr) samples and some reported Cr^3+^-activated NIR phosphors with emission over 800 nm.

| **NIR Phosphors** | **Emission peak (nm)** | **IQE (%)** | **PL intensty@423 K (%)** | **References** |
| --- | --- | --- | --- | --- |
| **CLMSO:Cr^3+^, flux** | 836 | 98.6 | 91.37 | This work |
| **CLMSO:Cr^3+^** | 836 | 82.5 | 89.80 | This work |
| **SLMSO:Cr^3+^** | 866 | 78.8 | 84.38 | This work |
| **InBO_3_** | 800 | 46.3 | 55 | [23] |
| **BaGe_4_O_9_** | 808 | 61.3 | 64 | [24] |
| **LiSrGaF_6_** | 813 | 76.8 | 61.62 | [25] |
| **Gd_3_MgScGa_2_SiO_12_** | 820 | 50 | 63 | [26] |
| **CaMgSi_2_O_6_** | 822 | 77.5 | 85 | [27] |
| **Na_2_CaHf_2_Ge_3_O_12_** | 830 | 79.2 | 71.2 | [28] |
| **RbAlF_4_** | 839 | 42.4 | Quenching | [29] |
| **Ca_4_ZrGe_3_O_12_** | 840 | 35 | 69 | [30] |
| **LiGaP_2_O_7_** | 846 | 47.8 | 51 | [31] |
| **Ga_4_GeO_8_** | 850 | 60 | 56 | [32] |
| **K_2_AlTi(PO_4_)_3_** | 857 | 76.4 | 20 | [33] |
| **LaSc_3_(BO_3_)_4_** | 871 | 23.29 | 30.20 | [34] |
| **K_4_Ga_3_Ta(PO_4_)_6_** | 873 | 27.4 | 35 | [35] |
| **LiIn_2_SbO_6_** | 892 | ~7 | ＜10 | [36] |
| **NaScGe_2_O_6_** | 895 | 40.22 | 20.5 | [37] |
| **NaInGe_2_O_6_** | 900 | 34 | ＜55 | [38] |
| **Cs_2_InCl_5_·H_2_O** | 910 | 75.1 | ＜52 | [39] |
| **Mg_4_Nb_2_O_9_** | 920 | 67.1 | 10.5 | [40] |
| **ZnTa_2_O_6_** | 935 | 24.5 | 49 | [41] |
| **LaTiTaO_6_** | 950 | 10.23 | 24.64 | [42] |
| **LiInF_4_** | 980 | 20 | ＜27 | [43] |
| **Cs_2_AgInCl_6_** | 1010 | 22.03 | ＜50 | [44] |
| **LiScW_2_O_8_** | 1069 | 42.90 | 10 | [45] |

**Table S9.** Quantum efficiency of CLMSO:Cr^3+^ phosphors with different H_3_BO_3_ contents.

| **H_3_BO_3_ contents** | **IQE (%)** | **AE (%)** | **EQE (%)** |
| --- | --- | --- | --- |
| **0wt%** | 82.5 | 38.1 | 31.4 |
| **1wt%** | 87.9 | 42.2 | 37.09 |
| **2wt%** | 98.6 | 35.0 | 34.5 |
| **3wt%** | 89.7 | 39.0 | 35.0 |
| **5wt%** | 82.7 | 35.5 | 29.4 |
| **8wt%** | 68.6 | 38.0 | 26.1 |

**Table S10.** Photoelectric properties of the fabricated pc-LED devices based on NIR phosphors (λ_em_ > 800 nm).

| **NIR Phosphors** | **NIR output power** | **Photoelectric efficiency** | **References** |
| --- | --- | --- | --- |
| **CLMSO:Cr^3+^, flux** | 36.71 mW@100 mA  98.43 mW@400 mA | 13.67%@100 mA | This work |
| **CLMSO:Cr^3+^, Yb^3+^** | 26.57 mW@100 mA  78.81 mW@400 mA | 10.32%@100 mA | This work |
| **CLMSO:Cr^3+^** | 25.19 mW@100 mA  68.77 mW@400 mA | 10.28%@100 mA | This work |
| **SLMSO:Cr^3+^** | 23.01 mW@100 mA  59.48 mW@400 mA | 9.22%@100 mA | This work |
| **LiScP_2_O_7_:Cr^3+^, Yb^3+^** | 36 mW@100 mA | 12.0%@100 mA | [46] |
| **Ca_2_LaZr_2_Ga_3_O_12_:Cr^3+^, Yb^3+^** | 32.2 mW@100 mA | 11.6%@100 mA | [47] |
| **InBO_3_:Cr^3+^** | 37.5 mW@120 mA | 10.42%@120 mA | [23] |
| **Ga_4_GeO_8_:Cr^3+^** | 28 mW@100 mA | 7.5%@100 mA | [32] |
| **RbAl_3_P_6_O_20_:Cr^3+^** | 27.9 mW@120 mA | 8.9%@120 mA | [46] |
| **Mg_3_Gd_2_Ge_3_O_12_:Cr^3+^** | 23.24 mW@100 mA | 8.45%@100 mA | [49] |
| **Sr_9_M(PO_4_)_7_:Cr^3+^** | ≈20 mW@100mA | 6.0%@100 mA | [50] |
| **Na_3_Al_2_(PO_4_)_2_F_3_:Cr^3+^** | ≈20 mW@100 mA | ≈9.0%@100 mA | [51] |
| **Gd_2_Mg_3_Ge_3_O_12_:Cr^3+^, Yb^3+^** | 17.21 mW@ 100mA | 5.76%@ 100 mA | [52] |
| **Ca_4_HfGe_3_O_12_:Cr^3+^** | 16.52 mW@100 mA | 5.92%@100 mA | [53] |
| **ZnTa_2_O_6_:Cr^3+^** | ≈15 mW@100 mA | ≈5.0%@100 mA | [41] |
| **CaTi_4_(PO_4_)_6_:Cr^3+^** | 14.4 mW@100 mA | 5.49%@100 mA | [54] |
| **RbAlF_4_:Cr^3+^** | 13.55 mW@100 mA | 4.51%@100 mA | [29] |
| **Mg_2_Al_4_Si_5_O_18_:Eu^2+^, Cr^3+^** | ≈10 mW@100 mA | 3.5%@100 mA | [55] |
| **KYbP_2_O_7_:Cr^3+^** | 8.8 mW@150 mA | 1.7%@150 mA | [56] |
| **ScF_3_:Cr^3+^** | ≈10 mW@100 mA | 3.19%@100 mA | [57] |
| **LiInF_4_:Cr^3+^** | 4.31 mW@100 mA | 1.60%@100 mA | [43] |

**References**

[1] J. Su, R. Pang, T. Tan, S. Wang, W. Yuan, J. Wang, X. Chen, H. Wu, C. Li, H. Zhang, *J. Mater. Chem. C* **2022**, 10, 10047-10057.

[2] Q. Cao, L. Li, J. Xie, Y. Zhang, W. Wang, Y. Pan, X. Wei, Y. Li, *J. Lumin.* **2023**, 266, 120314.

[3] H. Li, Y. Niu, A. A. Haider, C. Liu, C. You, H. Zhang, H. Jiang, J. Li, Y. Huang, S. Wang, D. Gao, S. Huang, J. Zhu, *Chem. Eng. J.* **2025**, 510, 161635.

[4] H. Gao, B. Devakumar, X. Huang, *Ceram. Int.* **2024**, 51, 8321-8328.

[5] M. Chen, H. Fan, Z. Lu, J. Song, X. Zhang, Q. Pang, P. Chen, L. Zhou, *Ceram. Int.* **2023**, 49, 15717-15725.

[6] L. Li, H. Yang, Y. Wang, F. Ling, X. Zhou, G. Xiang, Z. Cao, S. Jiang, Z. Yang, Y. Hua, *Ceram. Int.* **2023**, 50, 9753-9761.

[7] J. Zhang, W. Zhao, X. Zhang, Y. Li, W. Zhang, H. Wen, J. Zhong, *J. Lumin.* **2023**, 255, 119581.

[8] M. Zhao, F. Zhao, S. Liu, C. Wang, Z. Song, Q. Liu, *ACS Applied Optical Materials* **2024**, 2, 795-803.

[9] M. X. Han, S. Y. Chen, J. Li, Z. X. Gao, Y. Zhang, Y. Shen, Y. Tian, D. G. Deng, *J. Alloys Compd.* **2024**, 973, 172927.

[10] Y. T. Zhaoqi Liu, Xiaohong Zhang, Nian Pan, Haiyan Shi, Pengbo Lyu, Changfu Xu, Lizhong Sun, *Ceram. Int.* **2025**, DOI: 10.1016/j.ceramint.2025.03.346.

[11] M. Zhao, S. Liu, F. Zhao, H. Cai, Z. Song, Q. Liu, *Inorg. Chem. Front.* **2022**, 9, 4602-4607.

[12] M. Zhao, S. Q. Liu, H. Cai, F. Y. Zhao, Z. Song, Q. L. Liu, *Sci. China Mater.* **2022**, 65, 748-756.

[13] M. Han, Z. Gao, Y. Zhang, S. Chen, J. Li, Y. Shen, Y. Tian, D. Deng, *J. Alloys Compd.* **2024**, 995, 174780.

[14] Y. Fan, H. Wu, Y. Li, Y. Hu, *Ceram. Int.* **2024**, 50, 28707-28714.

[15] Y. Wang, Y. Sun, Z. Xu, X. Xing, M. Shang, *Inorg. Chem.* **2024**, 63, 8899-8907.

[16] F. Xiao, H. Xie, C. Xie, R. Yi, Y. Zeng, H. Yuan, *J. Lumin.* **2023**, 255, 119588.

[17] Y. Fan, H. Wu, Y. Jin, Y. Li, Y. Hu, *J. Lumin.* **2025**, 283, 121262.

[18] X. Wang, Y. Zhao, M. Yin, T. Zhou, R.-J. Xie, *J. Phys. Chem. C* **2023**, 127, 22799-22807.

[19] H. Suo, Y. Wang, X. Q. Zhao, X. Zhang, L. P. Li, K. W. Guan, W. G. Ding, P. L. Li, Z. J. Wang, F. Wang, *Laser Photonics Rev.* **2022**, 16, 2200012.

[20] H. Zeng, T. Zhou, L. Wang, R.-J. Xie, *Chem. Mater.* **2019**, 31, 5245-5253.

[21] G. Guo, Q. Xi, T. Yin, J. Nie, Y. Zhang, L. Guan, Z. Liu, F. Wang, X. Li, *J. Alloys Compd.* **2023**, 965, 171459.

[22] Y. Zhao, X. Wang, Q. Wang, J. Xu, Y. Chen, X. Tang, T. Zhou, R.-J. Xie, *J. Mater. Chem. C* **2024**, 12, 10532-10539.

[23] Z. S. Sun, Q. X. Ning, W. Y. Zhou, J. B. Luo, P. C. Chen, L. Y. Zhou, Q. Pang, X. G. Zhang, *Ceram. Int.* **2021**, 47, 13598-13603.

[24] Y. Tang, J. Yang, Q. Mao, Y. Ding, G. Zheng, L. Pei, J. Zhong, *J. Mater. Chem. C* **2024**, 12, 3980-3987.

[25] D. Wu, Y. Li, Y. Liao, X. Pan, S. Liu, W. Zou, J. Peng, X. Ye, *Dalton Trans.* **2023**, 52, 12526-12533.

[26] L. Jiang, X. Jiang, J. Xie, H. Sun, L. Zhang, X. Liu, Z. Bai, G. Lv, Y. Su, *J. Alloys Compd.* **2022**, 920, 165912.

[27] L. M. Fang, Z. D. Hao, L. L. Zhang, H. Wu, H. J. Wu, G. H. Pan, J. H. Zhang, *Mater. Res. Bull.* **2022**, 149, 111725.

[28] Z. Liao, Y. Li, J. Zhong, *Opt. Lett.* **2025**, 50, 634-637.

[29] H. Deng, X. Han, J. Wang, D. Liu, B. Zou, *J. Alloys Compd.* **2025**, 1014.

[30] J. M. Xiang, J. M. Zheng, X. Q. Zhao, X. Zhou, C. H. Chen, M. K. Jin, C. F. Guo, *Mater. Chem. Front.* **2022**, 6, 440-449.

[31] C. X. Yuan, R. Y. Li, Y. F. Liu, L. L. Zhang, J. H. Zhang, G. Leniec, P. Sun, Z. H. Liu, Z. H. Luo, R. Dong, J. Jiang, *Laser Photonics Rev.* **2021**, 15, 2100227.

[32] L. Yao, Q. Shao, M. Shi, T. Shang, Y. Dong, C. Liang, J. He, J. Jiang, *Adv. Opt. Mater.* **2021**, 10, 2102229.

[33] L. Chen, J. Zhong, *ACS Appl. Mater. Interfaces* **2024**, 16, 41119-41126.

[34] T. Y. Gao, W. D. Zhuang, R. H. Liu, Y. H. Liu, C. P. Yan, X. X. Chen, *Cryst. Growth Des.* **2020**, 20, 3851-3860.

[35] L. Jiang, D. Yang, L. Zhang, J. Wang, J. Zhang, W. Jiang, G. Li, H. Yu, W. Si, Z. Shi, Z. Zhang, Y. Su, *J. Alloys Compd.* **2024**, 1010, 177826.

[36] D. Liu, G. Li, P. Dang, Q. Zhang, Y. Wei, H. Lian, M. Shang, C. C. Lin, J. Lin, *Angew. Chem. Int. Ed. Engl.* **2021**, 60, 14644-14649.

[37] X. Zhou, W. Geng, J. Li, Y. Wang, J. Ding, Y. Wang, *Adv. Opt. Mater.* **2020**, 8, 1902003.

[38] W. Zhou, J. Luo, J. Fan, H. Pan, S. Zeng, L. Zhou, Q. Pang, X. Zhang, *Ceram. Int.* **2021**, 47, 25343-25349.

[39] L. Yu, Y. Wang, X. Xing, M. Shang, *J. Mater. Chem. C* **2024**, 12, 13021-13028.

[40] Q. Ding, J. Wu, D. Yu, X. Han, Y. Zhou, T. Shen, Y. Ma, S. Zhuang, D. Zhang, *J. Mater. Chem. C* **2024**, 12, 2184-2193.

[41] S. He, P. Li, Y. Ren, G. Wei, Y. Wang, Y. Yang, R. Li, J. Li, Y. Shi, X. Shi, Z. Wang, *Inorg. Chem.* **2022**, 61, 11284-11292.

[42] Y. Yang, Z. Lu, H. Fan, M. Chen, L. Shen, X. Zhang, Q. Pang, J. Chen, P. Chen, L. Zhou, *Inorg. Chem.* **2023**, 62, 3601-3608.

[43] L. Song, S. Liang, W. Nie, X. He, J. Hu, F. Lin, H. Zhu, *Inorg. Chem.* **2023**, 62, 11112-11120.

[44] F. Zhao, Z. Song, J. Zhao, Q. Liu, *Inorg. Chem. Front.* **2019**, 6, 3621-3628.

[45] J. Liu, D. Sun, Z. Lyu, S. Shen, S. Wei, L. Zhou, X. Zhang, H. Hu, H. You, *Adv. Opt. Mater.* **2024**, 12, 2401883.

[46] L. Q. Yao, Q. Y. Shao, S. Y. Han, C. Liang, J. H. He, J. Q. Jiang, *Chem. Mater.* **2020**, 32, 2430-2439.

[47] Y. Liu, S. He, D. Wu, X. Dong, W. Zhou, *ACS Appl. Electron. Mater.* **2022**, 4, 643-650.

[48] X. Wu, D. Huang, Q. Lin, L. Han, W. You, J. Zhu, X. Ye, *Inorg. Chem.* **2025**, 64, 3476-3484.

[49] C. Li, M. Sójka, J. Zhong, J. Brgoch, *Dalton Trans.* **2023**, 52, 12892-12898.

[50] F. Zhao, H. Cai, Z. Song, Q. Liu, *Chem. Mater.* **2021**, 33, 3621-3630.

[51] H. Wu, S. Liang, W.-X. You, L. Liu, Y. Guo, S. Wang, L. Song, Z. Wang, H. Zhu, *J. Mater. Chem. C* **2024**, 12, 4825-4834.

[52] C. Lyu, D. Sun, Z. Lyu, S. Shen, P. Luo, Z. Yue, Z. Lu, L. Zhou, H. You, *Mater. Today Chem.* **2024**, 36, 101963.

[53] Z. Liao, J. Zhong, C. Li, H. Jiang, W. Zhao, Phys. *Chem. Chem. Phys.* **2023**, 25, 15452-15462.

[54] B. Yan, J. Wen, Y. Zhou, Q. Sha, X. Huang, Z. Dong, C. Wang, Q. Wang, L. Ning, C.-K. Duan, *Ceram. Int.* **2024**, 51, 11018-11025.

[55] X. Zou, H. Zhang, W. Li, M. Zheng, M. S. Molokeev, Z. Xia, Y. Zheng, Q. Li, Y. Liu, X. Zhang, B. Lei, *Adv. Opt. Mater.* **2022**, 10, 2200882.

[56] R. Shi, S. Miao, Y. Zhang, X. Lv, D. Chen, Y. Liang, *J. Mater. Chem. C* **2023**, 11, 2748-2755.

[57] Q. Lin, Q. Wang, M. Liao, M. Xiong, X. Feng, X. Zhang, H. Dong, D. Zhu, F. Wu, Z. Mu, *ACS Appl. Mater. Interfaces* **2021**, 13, 18274-18282.
